# Supplementary material for: PROST: quantitative identification of spatially variable genes and domain detection in spatial transcriptomics
Source: Nat Commun. 2024 Jan 18;15:600. doi: 10.1038/s41467-024-44835-w (PMC10796707; doi:10.1038/s41467-024-44835-w)
Supplement: Supplementary file 1 — Supplementary information [file 41467_2024_44835_MOESM1_ESM.pdf]

Supplementary Information

PROST: quantitative identification of spatially variable genes and domain detection in spatial transcriptomics

Yuchen Liang[1], Guowei Shi[2], Runlin Cai[1], Yuchen Yuan[2], Ziying Xie[2], Long Yu[1], Yingjian Huang[1], Qian Shi[1], Lizhe Wang[3], Jun Li[3], Zhonghui Tang[2]

[1]School of Geography and Planning, Sun Yat-sen University, Guangzhou, 510275, China

[2]Zhongshan School of Medicine, Sun Yat-sen University, Guangzhou, 510080, China

[3]School of Computer Science, China University of Geosciences, Wuhan, 430078, China

Correspondence to:

Jun Li (lijuncug@cug.edu.cn) and Zhonghui Tang (tangzhh99@mail.sysu.edu.cn)

Supplementary Note 1. Measuring spatial autocorrelation using Moran's *I* and Geary's *C* statistics. To quantify the degree of spatial autocorrelation of gene expressions (i.e. gene expressions at nearby locations may get closer values than that farther apart), we applied two commonly used statistics Moran's *I* and Geary's *C* to calculate the spatial autocorrelation of gene expressions. Respectively, the Moran's *I* and Geary's *C* statistic are calculated as:

$$\text{Moran's } I = \frac{N}{W} \frac{\sum_i \sum_j \left[w_{ij} (x_i - \bar{x}) (x_j - \bar{x}) \right]}{\sum_i (x_i - \bar{x})^2}$$

$$\text{Geary's } C = \frac{N-1}{2W} \frac{\sum_i \sum_j \left[w_{ij} (x_i - x_j)^2 \right]}{\sum_i (x_i - \bar{x})^2}$$

where x_i and x_j are the expressions of spot i and spot j, $\bar{x}$ is the mean expression of the gene, N is the number of spots, and W is the sum of w_{ij}. Here, w_{ij} is the spatial weight between spot i and spot j, calculated based on the spatial coordinates of each spot. Noticeably, the value of Moran's *I* ranges from –1 to 1, where a value close to 1 indicates an apparent spatial pattern, while a value close to 0 indicates a random spatial expression. The value of Geary's *C* ranges from 0 to 2, where 0, 1, and 2 represents a perfect positive autocorrelation, no autocorrelation, or a perfect negative autocorrelation, respectively.

Supplementary Table 1. Methods compared with PROST in this study.

Method	Version	Platform	URL	Reference
SCANPY	1.8.2	Python	https://github.com/scverse/scanpy	[1]
stLearn	0.4.7	Python	https://github.com/BiomedicalMachineLearning/stLearn	[2]
Giotto	1.1.2	R	https://github.com/RubD/Giotto	[3]
BayesSpace	1.6.0	R	https://github.com/edward130603/BayesSpace	[4]
SpaGCN	1.2.5	Python	https://github.com/jianhuupenn/SpaGCN	[5]
SpaceFlow	1.0.4	Python	https://github.com/hongleir/SpaceFlow	[6]
STAGATE	1.0.1	Python	https://github.com/zhanglabtools/STAGATE	[7]
BASS	1.1.0.016	R	https://github.com/zhengli09/BASS	[8]
Seurat	4.1.0	R	https://satijalab.org/seurat/	[9]
SpatialDE	1.1.3	Python	https://github.com/Teichlab/SpatialDE	[10]
SPARK-X	1.1.1	R	https://xzhoulab.github.io/SPARK/	[11]
scGCO	1.1.2	Python	https://github.com/WangPeng-Lab/scGCO	[12]
SINFONIA	0.0.3	Python	https://github.com/BioX-NKU/SINFONIA	[13]

Supplementary Table 2. Published datasets analyzed in this study.

Platform	Tissue	Section	Dataset dimensions	Reference
10x Visium	Human Dorsolateral prefrontal cortex (DLPFC)	151507	4,226spots × 33,538genes	[14]
		151508	4,384spots × 33,538genes	
		151509	4,789spots × 33,538genes	
		151510	4,634spots × 33,538genes	
		151669	3,661spots × 33,538genes	
		151670	3,498spots × 33,538genes	
		151671	4,110spots × 33,538genes	
		151672	4,015spots × 33,538genes	
		151673	3,639spots × 33,538genes	
		151674	3,673spots × 33,538genes	
		151675	3,592spots × 33,538genes	
		151676	3,460spots × 33,538genes	
	Adult Mouse Brain	Brain Section	2,698spots × 31,053genes	10x Visium demo
		Section 1 (Coronal)	2,603spots × 32,285genes	
		Section 1 (Sagittal-Anterior)	2,695spots × 32,285genes	
		Section 1 (Sagittal-Posterior)	3,353spots × 31,053genes	
	Breast Cancer Block	A Section 1	3,798spots × 36,601genes	
	Human Lymph Node		4,039spots × 33,538genes	
	Mouse Kidney		3,124spots × 19,465genes	
ST sequencing	adult human heart	LV	61spots × 1,830genes	[15]
		LV	60spots × 2,252genes	
		RAA	282spots × 7,275genes	
		RAA	292spots × 6,763genes	
		LV	55spots × 1,702genes	
		LV	52spots × 1,721genes	
		LV	107spots × 1,294genes	
		LV	85spots × 2,167genes	
		RAA	534spots × 8,552genes	
		RAA	365spots × 7,494genes	
Slide-seq	Cerebellum	Puck_180430_6	25,551spots × 17,729genes	[16]
	Sagittal Cerebellum	Puck_180819_11	1,159spots × 917genes	
	Sagittal Cortex	Puck_180819_19	9,699spots × 2,555genes	
	Coronal Hippocamus	Puck_180413_7	12,282spots × 3,235genes	
	Liver	Puck_180803_8	17,712spots × 2,238genes	
	Kidney	Puck_180528_23	23,006spots × 4,650genes	

osmFISH	Somatosensory Cortex		5,328spots × 33genes	[17]
Stereo-seq	Mouse Olfactory Bulb		19,109spots × 27,106genes	[18]
Slide-seq V2	Mouse Olfactory Bulb	Puck_200127_15	21,724spots × 21,217genes	[19]
SeqFISH	Mouse embryo	Embryo 1	19,451spots × 351genes	[20]
		Embryo 2	14,891spots × 351genes	
		Embryo 3	23,194spots × 351genes	
Simulated data	Somatosensory Cortex based on osmFISH		4,839spots × 33genes	[21]
	Mouse Brain based on 10x Visium	Sagittal-Anterior	2,696spots × 31,053genes	

Supplementary Table 3. Comparison of the domain segmentation performance. The adjusted rand indexes (ARIs) between PROST and other methods on the DLPFC dataset across twelve sections were used for the evaluation. The top ARI for each section is highlighted in bold.

**Section ID**	**PROST**	**SCANPY**	**stLearn**	**Bayes-Space**	**SpaGCN**	**HMRF**	**Space-Flow**	**STAGATE**	**BASS**
151507	0.508	0.288	0.464	0.457	0.466	0.452	0.469	0.368	**0.525**
151508	**0.525**	0.196	0.302	0.484	0.361	0.360	0.393	0.328	0.494
151509	0.460	0.209	0.422	0.433	0.510	**0.521**	0.303	0.412	0.402
151510	**0.488**	0.195	0.265	0.379	0.439	0.351	0.398	0.317	0.476
151669	0.382	0.203	0.337	**0.427**	0.282	0.294	0.302	0.291	0.391
151670	0.323	0.282	0.187	**0.430**	0.373	0.281	0.256	0.270	0.303
151671	0.506	0.247	0.505	0.473	0.532	0.412	0.359	0.382	**0.534**
151672	0.591	0.210	0.337	**0.716**	0.539	0.306	0.358	0.467	0.430
151673	0.510	0.261	0.363	**0.550**	0.369	0.318	0.432	0.466	0.528
151674	**0.474**	0.317	0.350	0.293	0.386	0.230	0.317	0.365	0.298
151675	0.453	0.291	0.388	0.352	0.459	0.298	0.316	0.307	**0.514**
151676	0.470	0.283	0.356	0.349	0.312	0.296	0.313	0.258	**0.578**
average	**0.474**	0.248	0.356	0.445	0.419	0.343	0.351	0.353	0.456
median	0.481	0.254	0.353	0.431	0.413	0.312	0.337	0.347	**0.485**

Supplementary Table 4. Comparison of the domain segmentation performance. The normalized mutual information (NMI) between PROST and other methods on the DLPFC dataset across twelve sections were used for the evaluation. The top NMI for each section is highlighted in bold.

**Section ID**	**PROST**	**SCANPY**	**stLearn**	**Bayes-Space**	**SpaGCN**	**HMRF**	**Space-Flow**	**STAGATE**	**BASS**
151507	0.624	0.365	0.613	0.658	0.587	0.554	0.575	0.531	**0.650**
151508	**0.669**	0.289	0.517	0.618	0.462	0.452	0.510	0.527	0.599
151509	**0.621**	0.338	0.608	0.602	0.615	0.607	0.491	0.601	0.605
151510	**0.629**	0.298	0.492	0.588	0.573	0.507	0.519	0.486	0.605
151669	0.538	0.274	0.505	**0.587**	0.398	0.377	0.415	0.433	0.572
151670	0.491	0.268	0.350	**0.548**	0.479	0.432	0.393	0.435	0.477
151671	0.641	0.249	0.575	0.600	0.641	0.510	0.462	0.485	**0.649**
151672	0.682	0.212	0.469	**0.703**	0.629	0.419	0.476	0.564	0.586
151673	0.666	0.373	0.542	**0.682**	0.538	0.452	0.539	0.612	0.652
151674	**0.575**	0.389	0.541	0.485	0.550	0.350	0.418	0.505	0.489
151675	0.584	0.348	0.563	0.599	0.555	0.451	0.462	0.414	**0.664**
151676	0.593	0.371	0.531	0.533	0.491	0.444	0.422	0.436	**0.686**
average	**0.610**	0.314	0.526	0.600	0.543	0.463	0.474	0.502	0.603
median	**0.622**	0.318	0.536	0.599	0.553	0.451	0.469	0.496	0.605

Supplementary Figure 1. Showing domain segmentation, UMAP visualizations, PAGA graphs and spatial expression patterns of SVGs using a 10x Visium human dorsolateral prefrontal cortex (DLPFC) dataset with section ID 151507. a, H&E image. **b**, Manual annotation. **c**, Domain segmentation by SCANPY, stLearn, HMRF, BayesSpace, SpaGCN, SpaceFlow, STAGATE, BASS, and PROST, respectively. **d**, UMAP visualizations and PAGA graphs colored by the manual annotation of spots, using a low-dimensional representation from SCANPY, stLearn, SpaceFlow, STAGATE, and PROST, respectively. **e**, Spatial expression patterns for the top-ranked SVGs detected by the PROST Index.

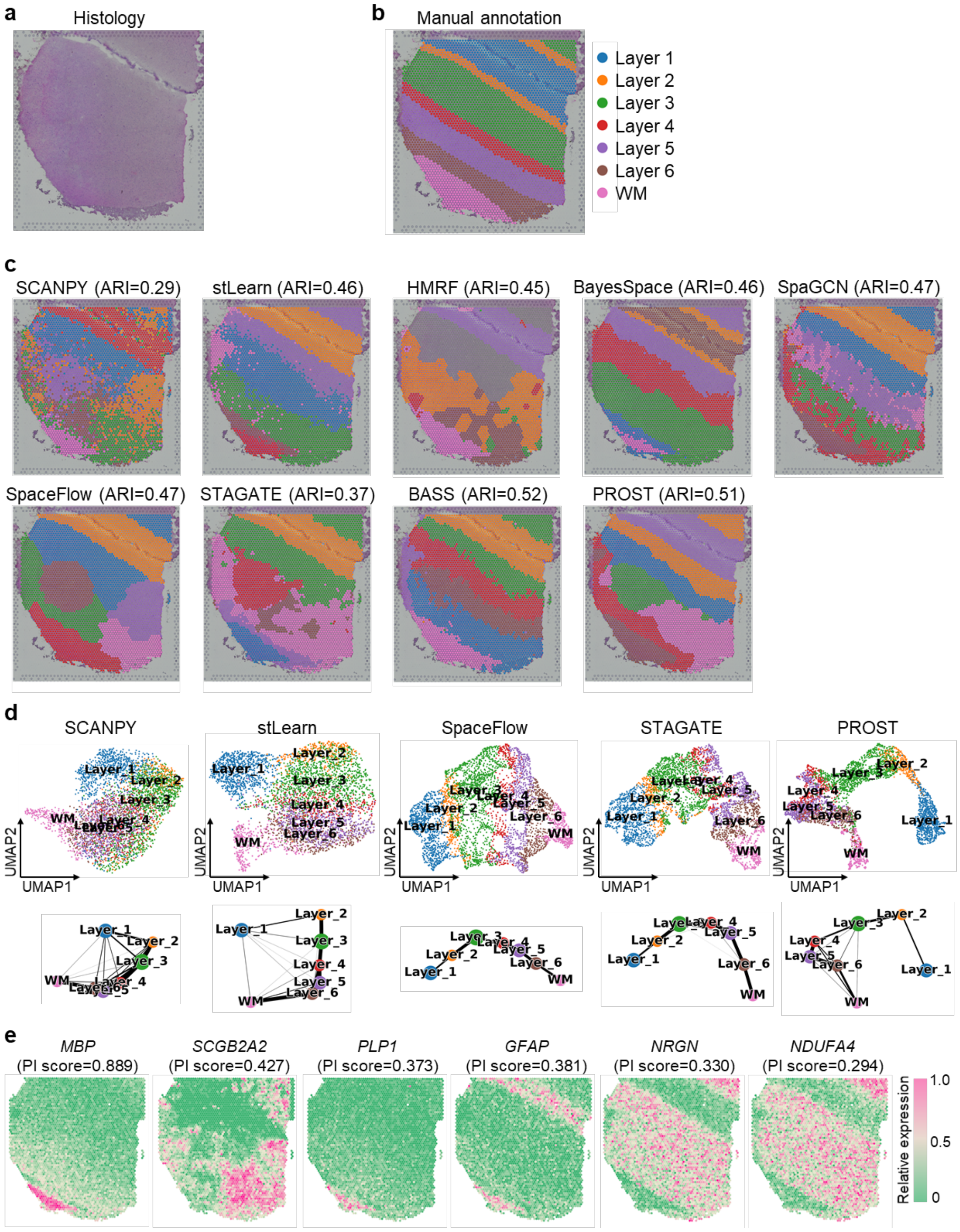

Supplementary Figure 2. Showing domain segmentation, UMAP visualizations, PAGA graphs and spatial pattern of SVGs using a 10x Visium DLPFC dataset with section ID 151508. a, H&E image. **b**, Manual annotation. **c**, Domain segmentation by SCANPY, stLearn, HMRF, BayesSpace, SpaGCN, SpaceFlow, STAGATE, BASS, and PROST, respectively. **d**, UMAP visualizations and PAGA graphs colored by the manual annotation of spots, using a low-dimensional representation from SCANPY, stLearn, SpaceFlow, STAGATE, and PROST, respectively. **e**, Spatial expression patterns for the top-ranked SVGs detected by the PROST Index.

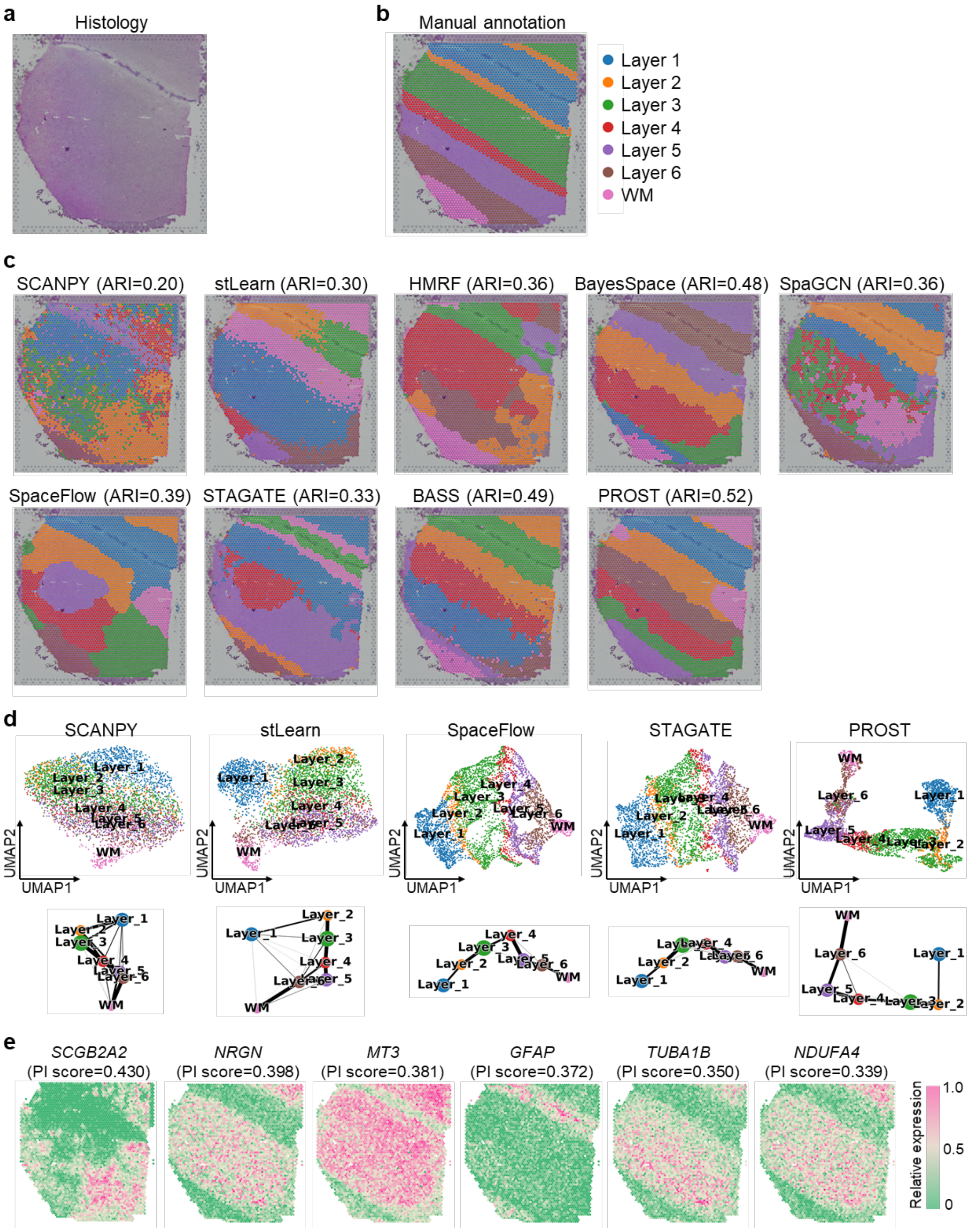

Supplementary Figure 3. Showing domain segmentation, UMAP visualizations, PAGA graphs and spatial pattern of SVGs using a 10x Visium DLPFC dataset with section ID 151509. a, H&E image. **b**, Manual annotation. **c**, Domain segmentation by SCANPY, stLearn, HMRF, BayesSpace, SpaGCN, SpaceFlow, STAGATE, BASS, and PROST, respectively. **d**, UMAP visualizations and PAGA graphs colored by the manual annotation of spots, using a low-dimensional representation from SCANPY, stLearn, SpaceFlow, STAGATE, and PROST, respectively. **e**, Spatial expression patterns for the top-ranked SVGs detected by the PROST Index.

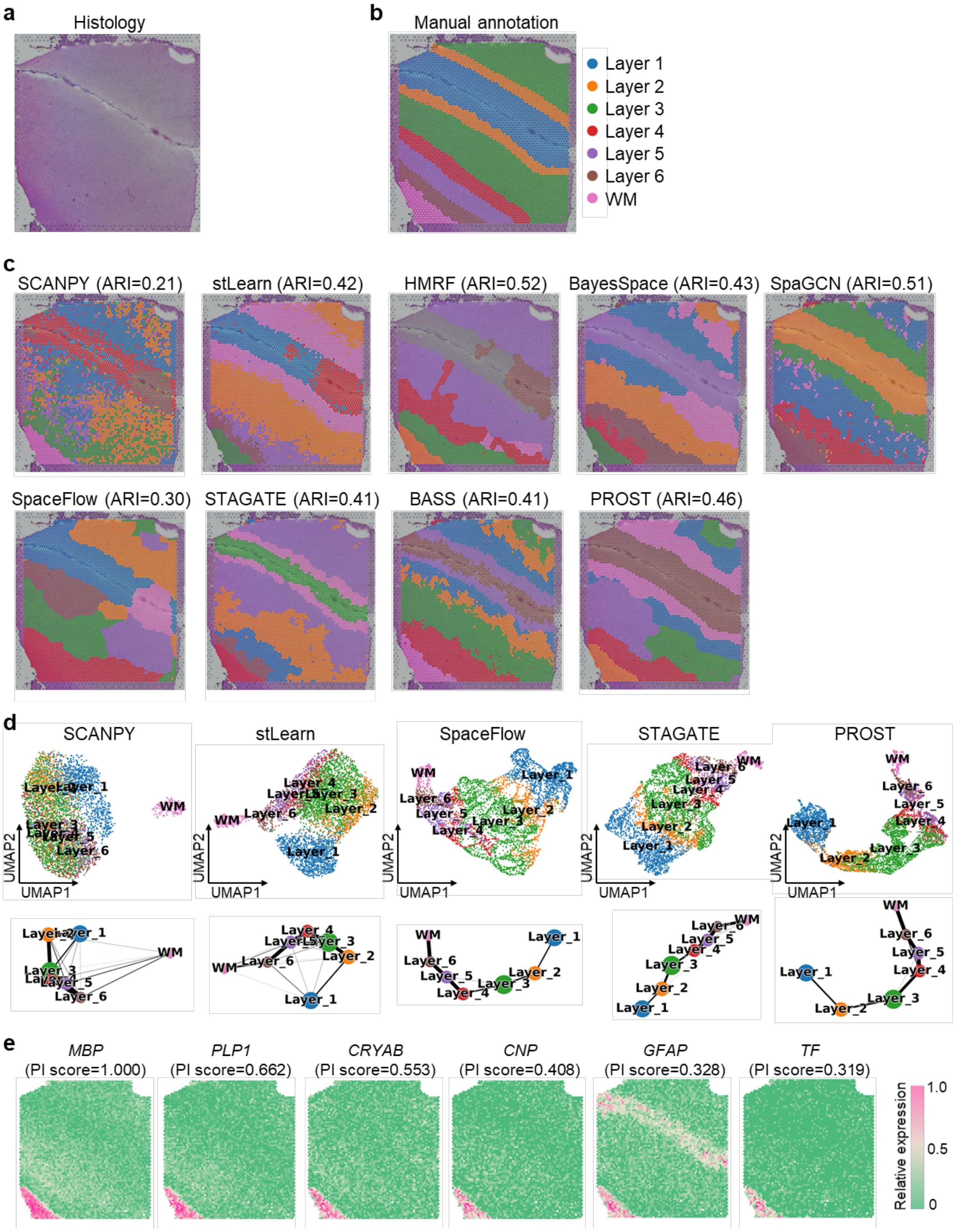

Supplementary Figure 4. Showing domain segmentation, UMAP visualizations, PAGA graphs and spatial pattern of SVGs using a 10x Visium DLPFC dataset with section ID 151510. a, H&E image. **b**, Manual annotation. **c**, Domain segmentation by SCANPY, stLearn, HMRF, BayesSpace, SpaGCN, SpaceFlow, STAGATE, BASS, and PROST, respectively. **d**, UMAP visualizations and PAGA graphs colored by the manual annotation of spots, using a low-dimensional representation from SCANPY, stLearn, SpaceFlow, STAGATE, and PROST, respectively. **e**, Spatial expression patterns for the top-ranked SVGs detected by the PROST Index.

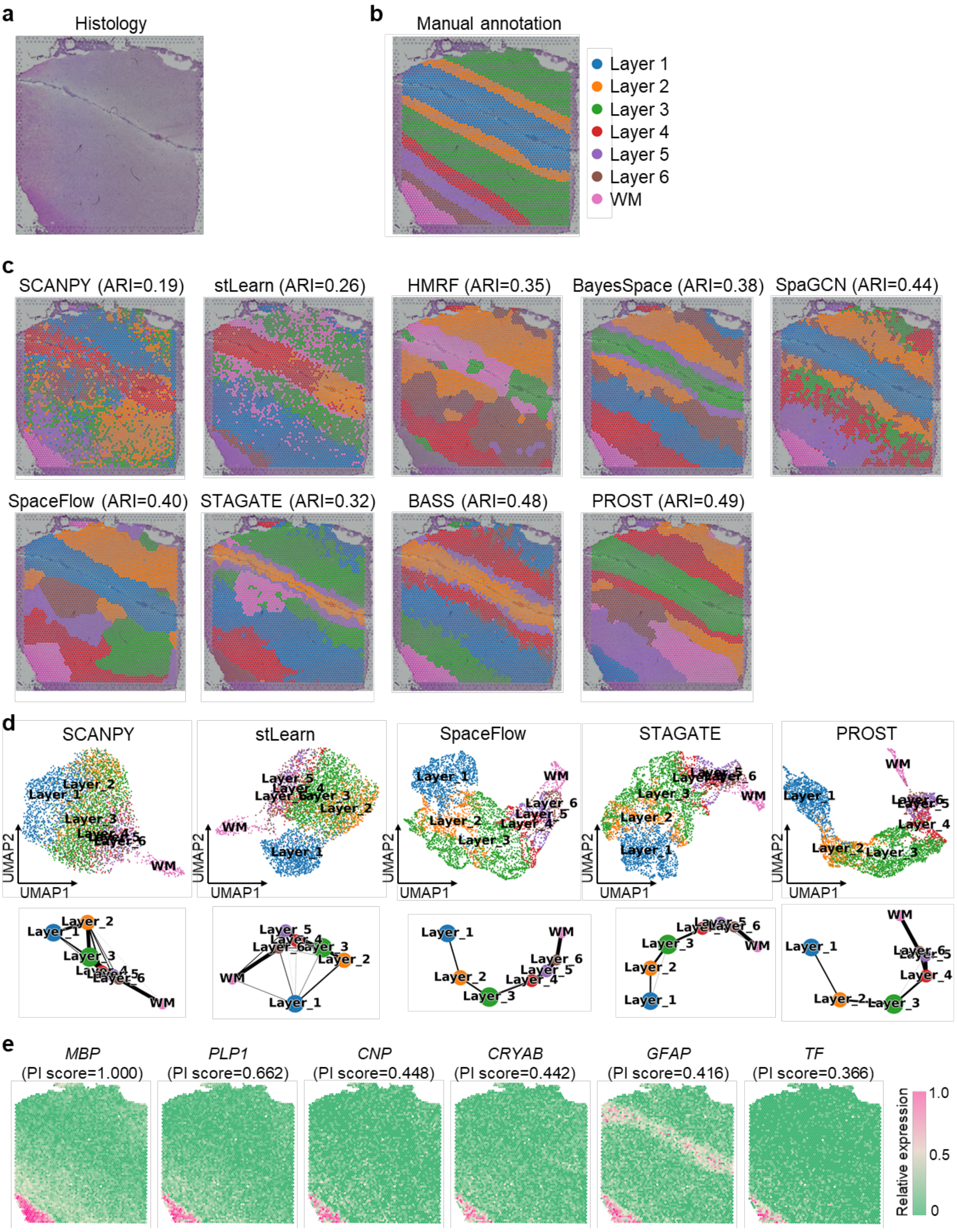

Supplementary Figure 5. Showing domain segmentation, UMAP visualizations, PAGA graphs and spatial pattern of SVGs using a 10x Visium DLPFC dataset with section ID 151669. a, H&E image. **b**, Manual annotation. **c**, Domain segmentation by SCANPY, stLearn, HMRF, BayesSpace, SpaGCN, SpaceFlow, STAGATE, BASS, and PROST, respectively. **d**, UMAP visualizations and PAGA graphs colored by the manual annotation of spots, using a low-dimensional representation from SCANPY, stLearn, SpaceFlow, STAGATE, and PROST, respectively. **e**, Spatial expression patterns for the top-ranked SVGs detected by the PROST Index.

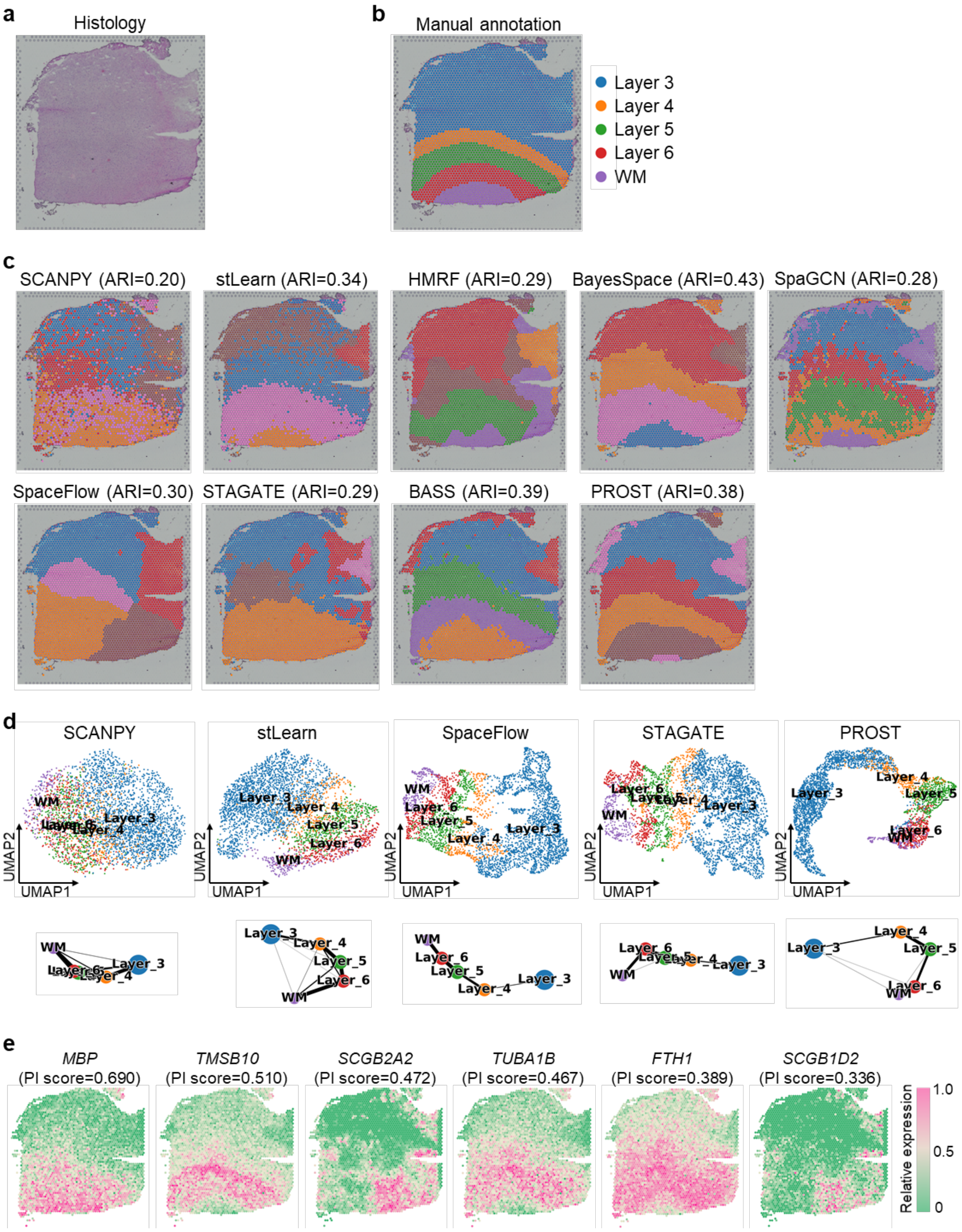

Supplementary Figure 6. Showing domain segmentation, UMAP visualizations, PAGA graphs and spatial pattern of SVGs using a 10x Visium DLPFC dataset with section ID 151670. a, H&E image. **b**, Manual annotation. **c**, Domain segmentation by SCANPY, stLearn, HMRF, BayesSpace, SpaGCN, SpaceFlow, STAGATE, BASS, and PROST, respectively. **d**, UMAP visualizations and PAGA graphs colored by the manual annotation of spots, using a low-dimensional representation from SCANPY, stLearn, SpaceFlow, STAGATE, and PROST, respectively. **e**, Spatial expression patterns for the top-ranked SVGs detected by the PROST Index.

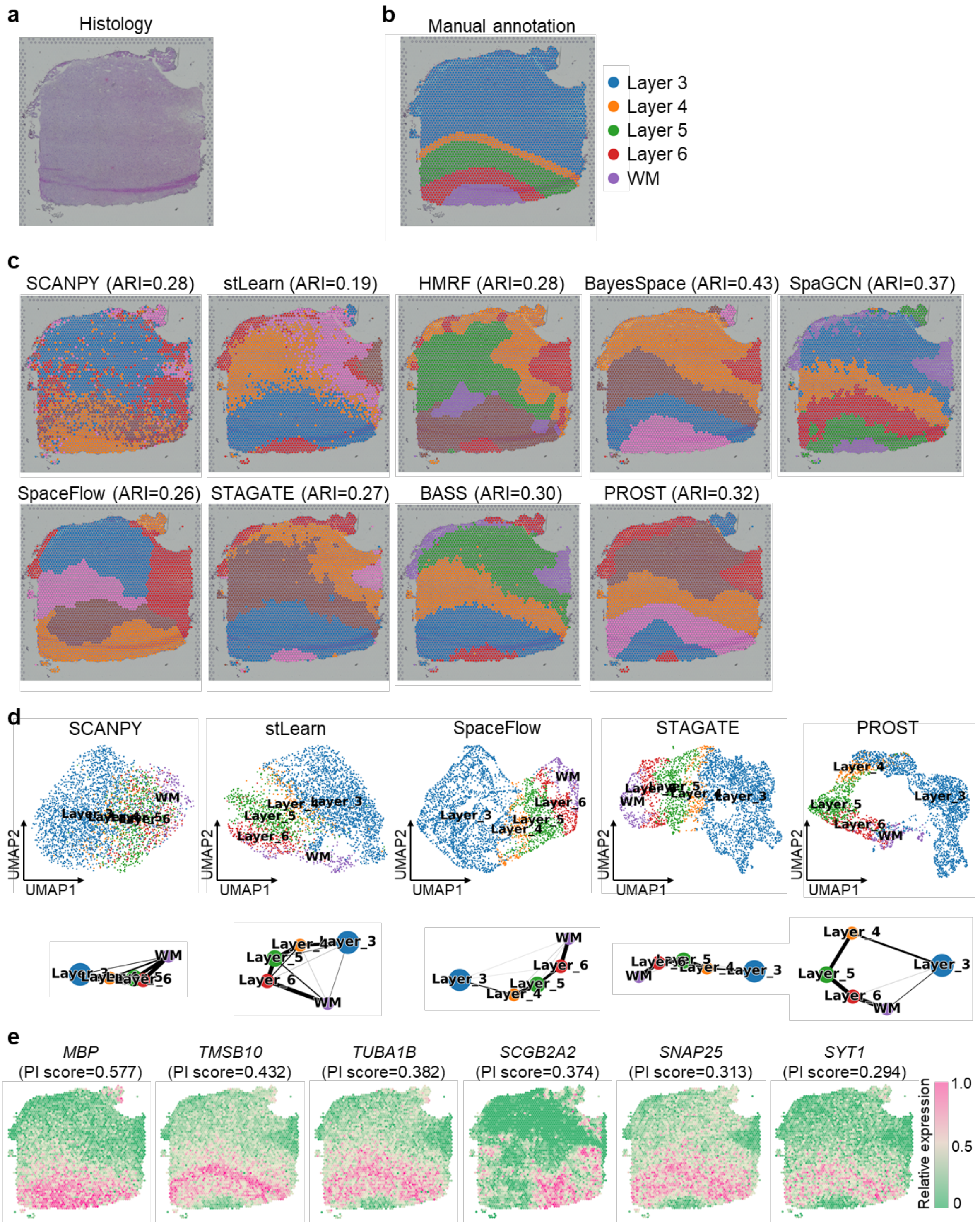

Supplementary Figure 7. Showing domain segmentation, UMAP visualizations, PAGA graphs and spatial pattern of SVGs using a 10x Visium DLPFC dataset with section ID 151671. a, H&E image. **b**, Manual annotation. **c**, Domain segmentation by SCANPY, stLearn, HMRF, BayesSpace, SpaGCN, SpaceFlow, STAGATE, BASS, and PROST, respectively. **d**, UMAP visualizations and PAGA graphs colored by the manual annotation of spots, using a low-dimensional representation from SCANPY, stLearn, SpaceFlow, STAGATE, and PROST, respectively. **e**, Spatial expression patterns for the top-ranked SVGs detected by the PROST Index.

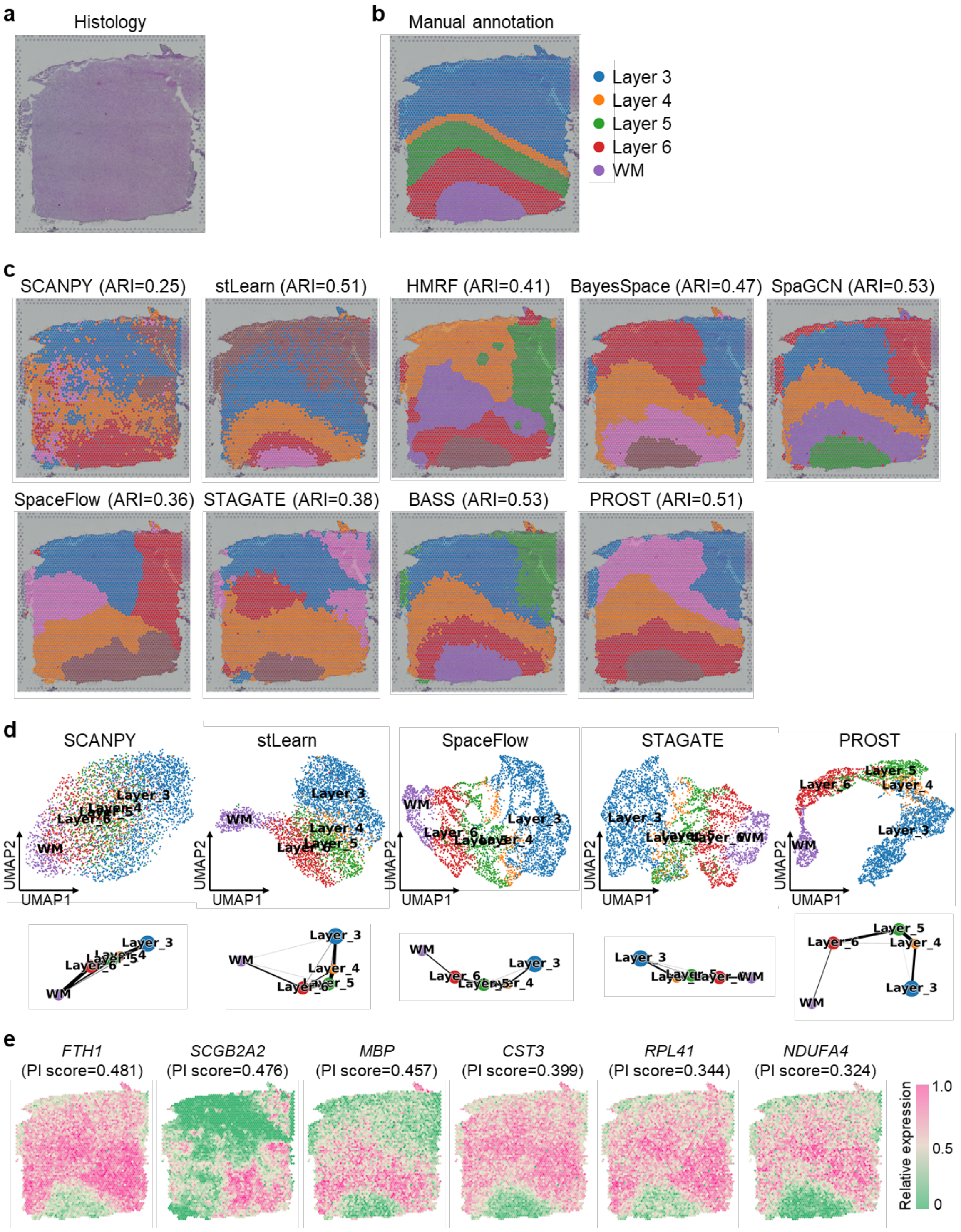

Supplementary Figure 8. Showing domain segmentation, UMAP visualizations, PAGA graphs and spatial pattern of SVGs using a 10x Visium DLPFC dataset with section ID 151673. a, H&E image. **b**, Manual annotation. **c**, Domain segmentation by SCANPY, stLearn, HMRF, BayesSpace, SpaGCN, SpaceFlow, STAGATE, BASS, and PROST, respectively. **d**, UMAP visualizations and PAGA graphs colored by the manual annotation of spots, using a low-dimensional representation from SCANPY, stLearn, SpaceFlow, STAGATE, and PROST, respectively. **e**, Spatial expression patterns for the top-ranked SVGs detected by the PROST Index.

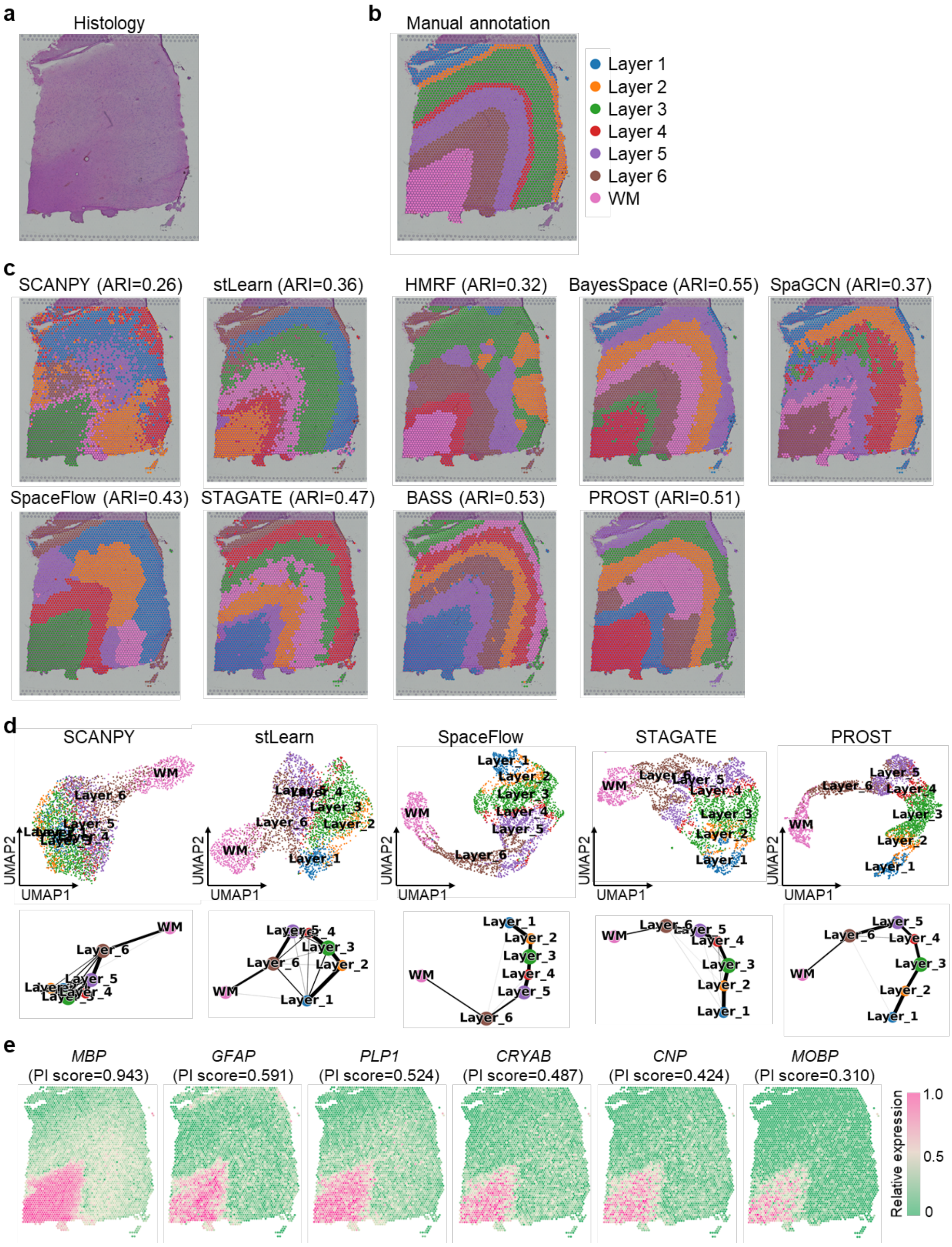

Supplementary Figure 9. Showing domain segmentation, UMAP visualizations, PAGA graphs and spatial pattern of SVGs using a 10x Visium DLPFC dataset with section ID 151674. a, H&E image. **b**, Manual annotation. **c**, Domain segmentation by SCANPY, stLearn, HMRF, BayesSpace, SpaGCN, SpaceFlow, STAGATE, BASS, and PROST, respectively. **d**, UMAP visualizations and PAGA graphs colored by the manual annotation of spots, using a low-dimensional representation from SCANPY, stLearn, SpaceFlow, STAGATE, and PROST, respectively. **e**, Spatial expression patterns for the top-ranked SVGs detected by the PROST Index.

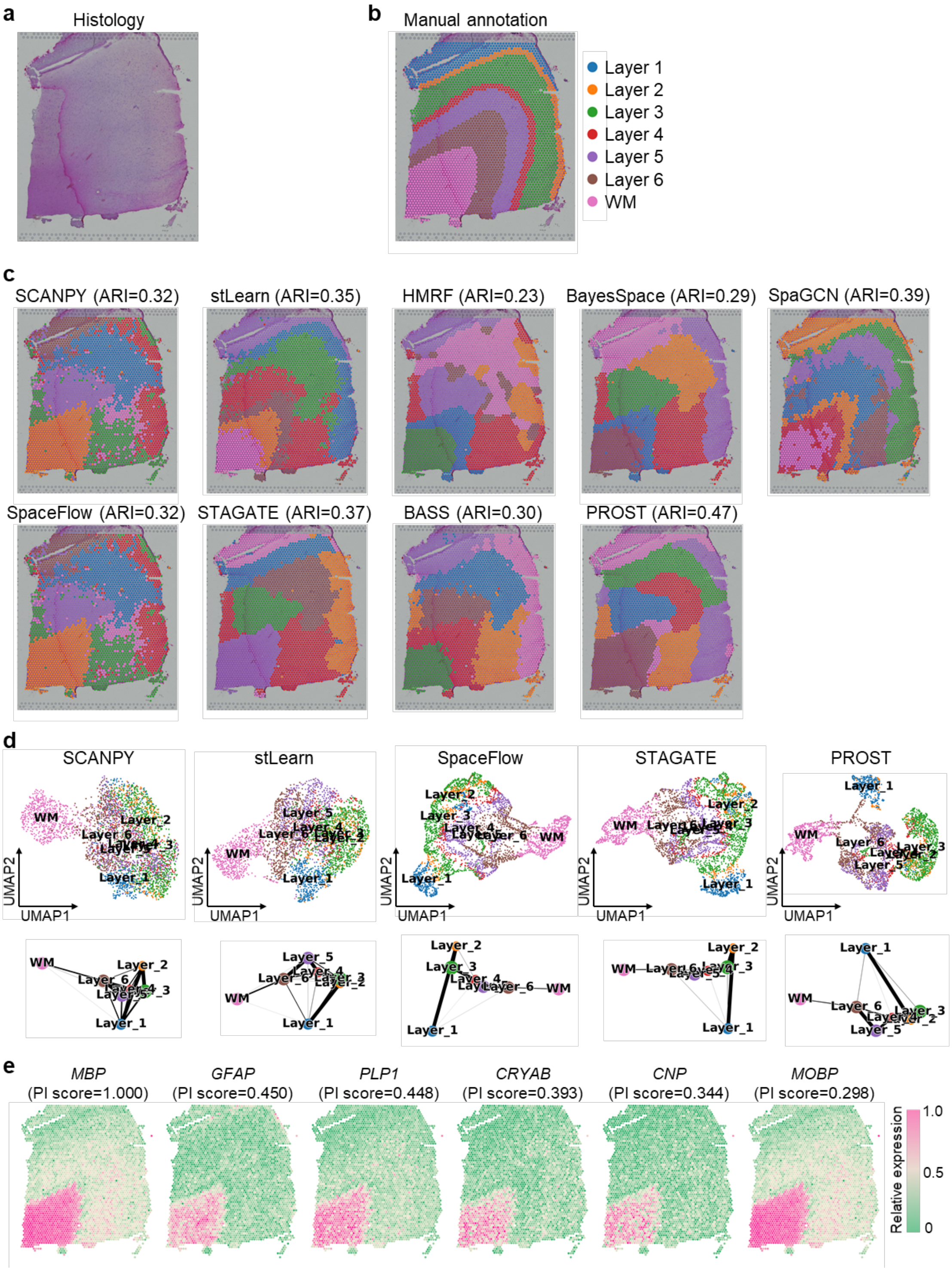

Supplementary Figure 10. Showing domain segmentation, UMAP visualizations, PAGA graphs and spatial pattern of SVGs using a 10x Visium DLPFC dataset with section ID 151675. a, H&E image. **b**, Manual annotation. **c**, Domain segmentation by SCANPY, stLearn, HMRF, BayesSpace, SpaGCN, SpaceFlow, STAGATE, BASS, and PROST, respectively. **d**, UMAP visualizations and PAGA graphs colored by the manual annotation of spots, using a low-dimensional representation from SCANPY, stLearn, SpaceFlow, STAGATE, and PROST, respectively. **e**, Spatial expression patterns for the top-ranked SVGs detected by the PROST Index.

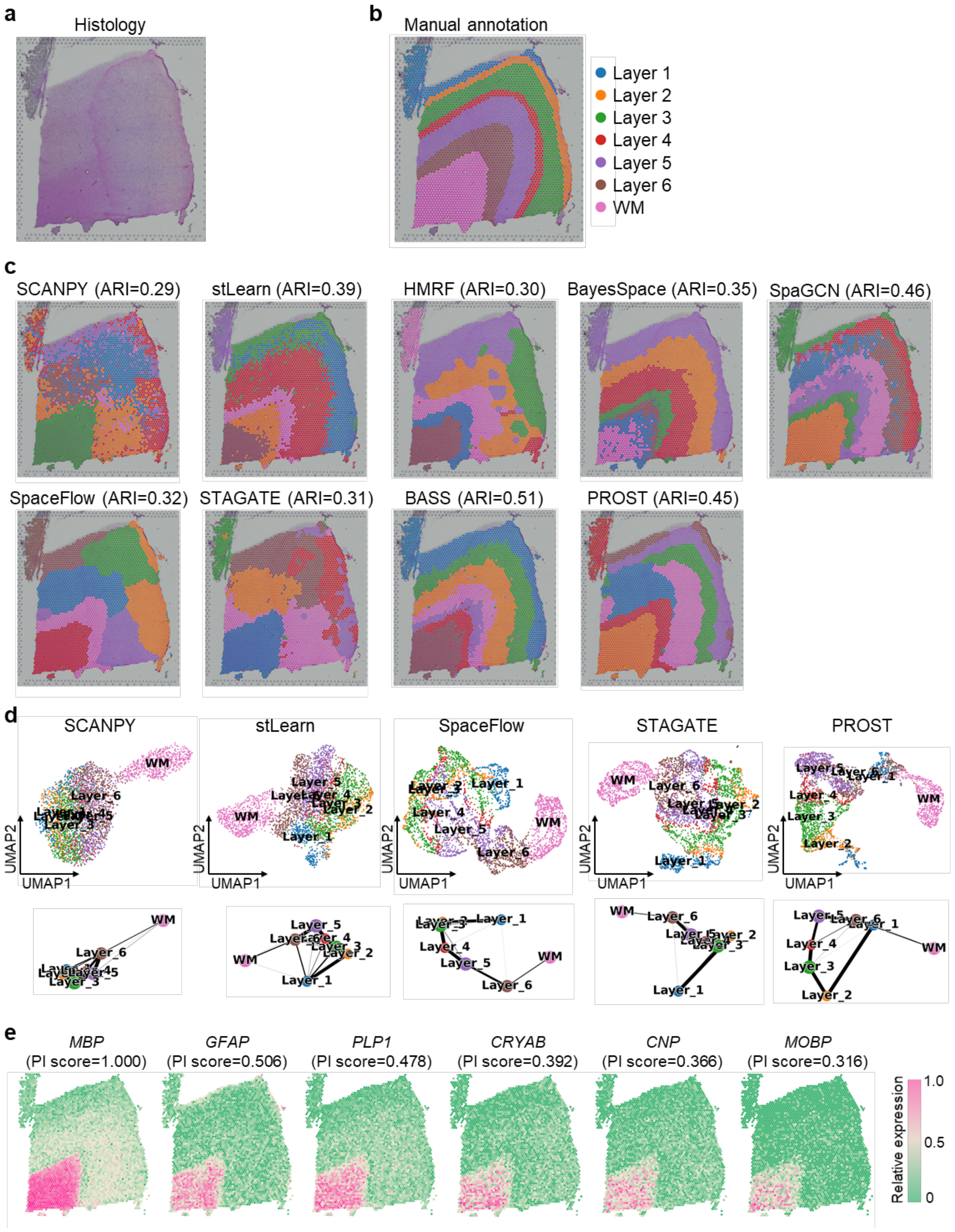

Supplementary Figure 11. Dispalying domain segmentation, UMAP visualizations, PAGA graphs and spatial pattern of SVGs using a 10x Visium DLPFC dataset with section ID 151676. a, H&E image. **b**, Manual annotation. **c**, Domain segmentation by SCANPY, stLearn, HMRF, BayesSpace, SpaGCN, SpaceFlow, STAGATE, BASS, and PROST, respectively. **d**, UMAP visualizations and PAGA graphs colored by the manual annotation of spots, using a low-dimensional representation from SCANPY, stLearn, SpaceFlow, STAGATE, and PROST, respectively. **e**, Spatial expression patterns for the top-ranked SVGs detected by the PROST Index.

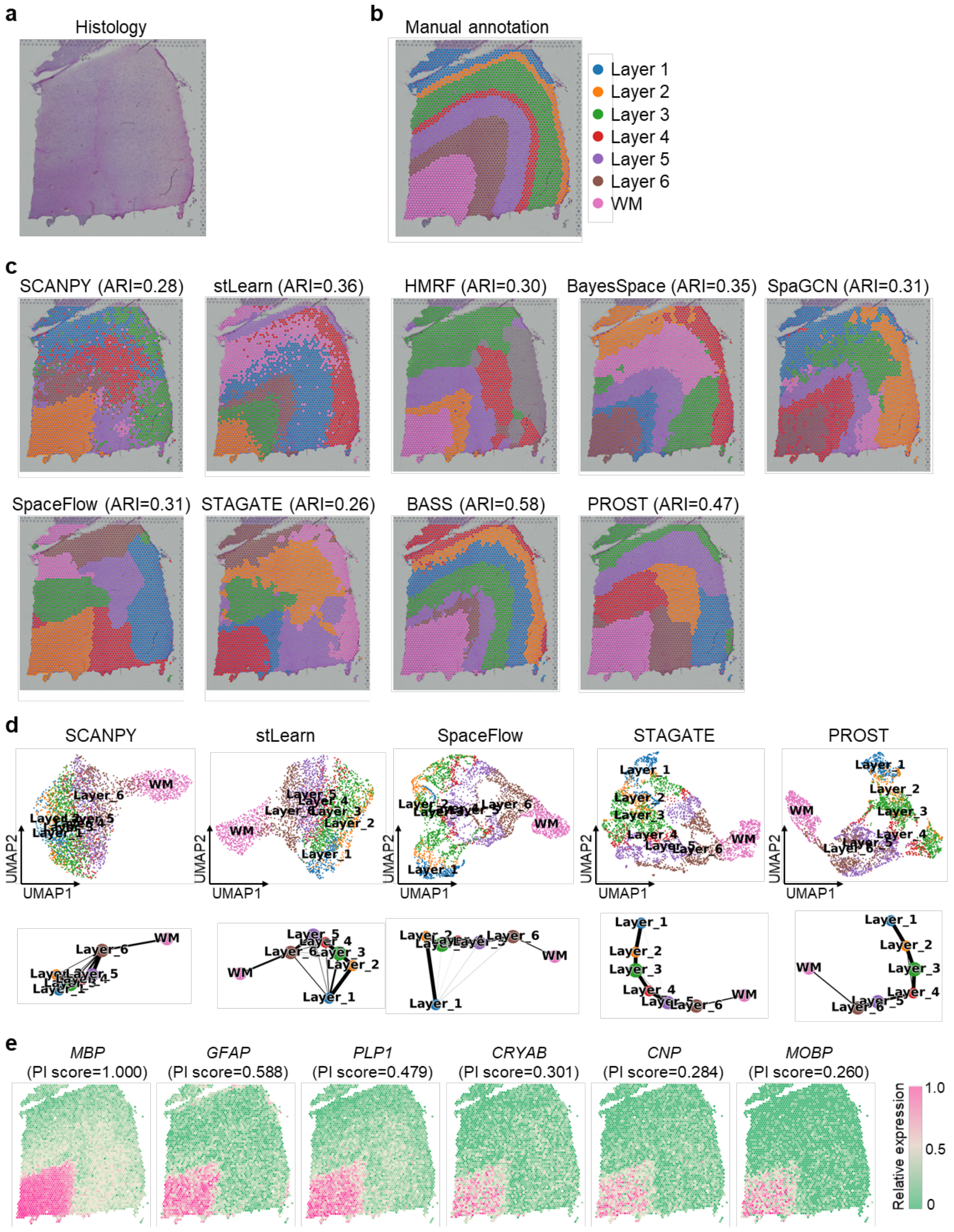

Supplementary Figure 12. PROST analysis using the 10x Visium Breast Cancer dataset. a, H&E image of the breast cancer tissue section. **b**, Manual annotation of breast cancer tissue section from SEDR[18]. **c**, Domain segmentation using PROST with 0.3, 1.0, and 1.4 resolution settings. **d**, UMAP visualizations colored by the manual annotation[18], using a low-dimensional representation from PROST with 0.3, 1.0, and 1.4 resolution settings. **e**, Spatial expression patterns for the top-ranked SVGs detected by the PROST Index.

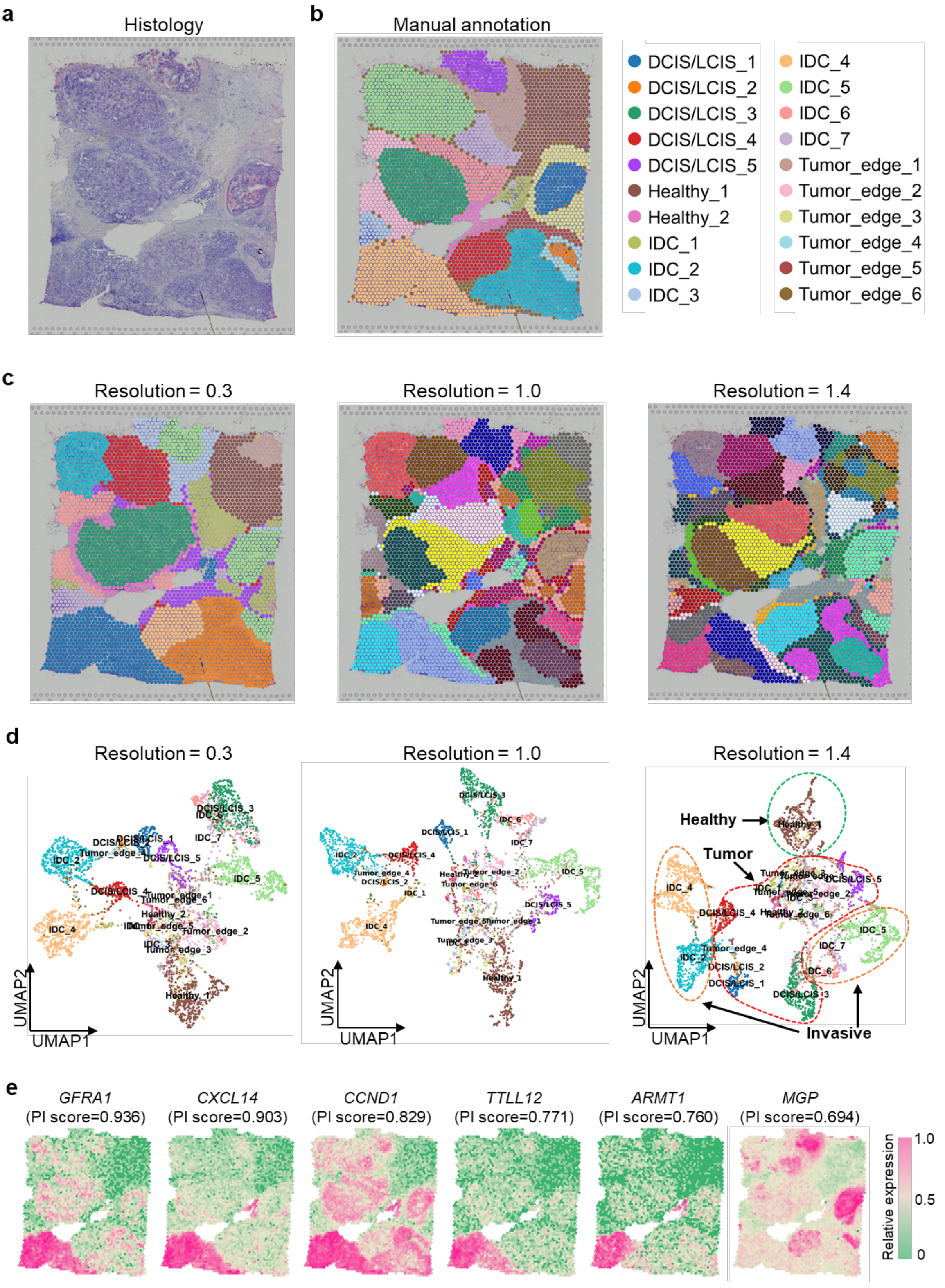

Supplementary Figure 13. PROST analysis on two 10X visium Adult Mouse Brain datasets. H&E image (**a**) and fluorescence image (**c**) of adult mouse brain tissue sections. **b & d**, Domain segmentations of the two 10x Visium Adult Mouse Brain datasets using PROST with 0.3, 0.8, and 1.4 resolution settings, respectively. **e**, The annotation of an adult mouse brain from the Allen Reference Atlas[22]. **f**, UMAP visualizations and PAGA graphs colored by the clusters of spots, using a low-dimensional representation from PROST with a resolution setting of 0.8, where the spots were annotated as Isocortex, olfactory areas, Hypothalamus (HY), Thalamus (TH) and Hippocampal formation (HPF) corresponding to the Allen Mouse Brain Atlas[22], illustrating better capability of PROST in learning low-dimensional representation. **g**, Zoomed-in view of domain segmentation by PROST at the mouse hippocampus region, compared with the Allen Mouse Brain Atlas[22]. The zoomed-in region is highlighted in the H&E image with a box.

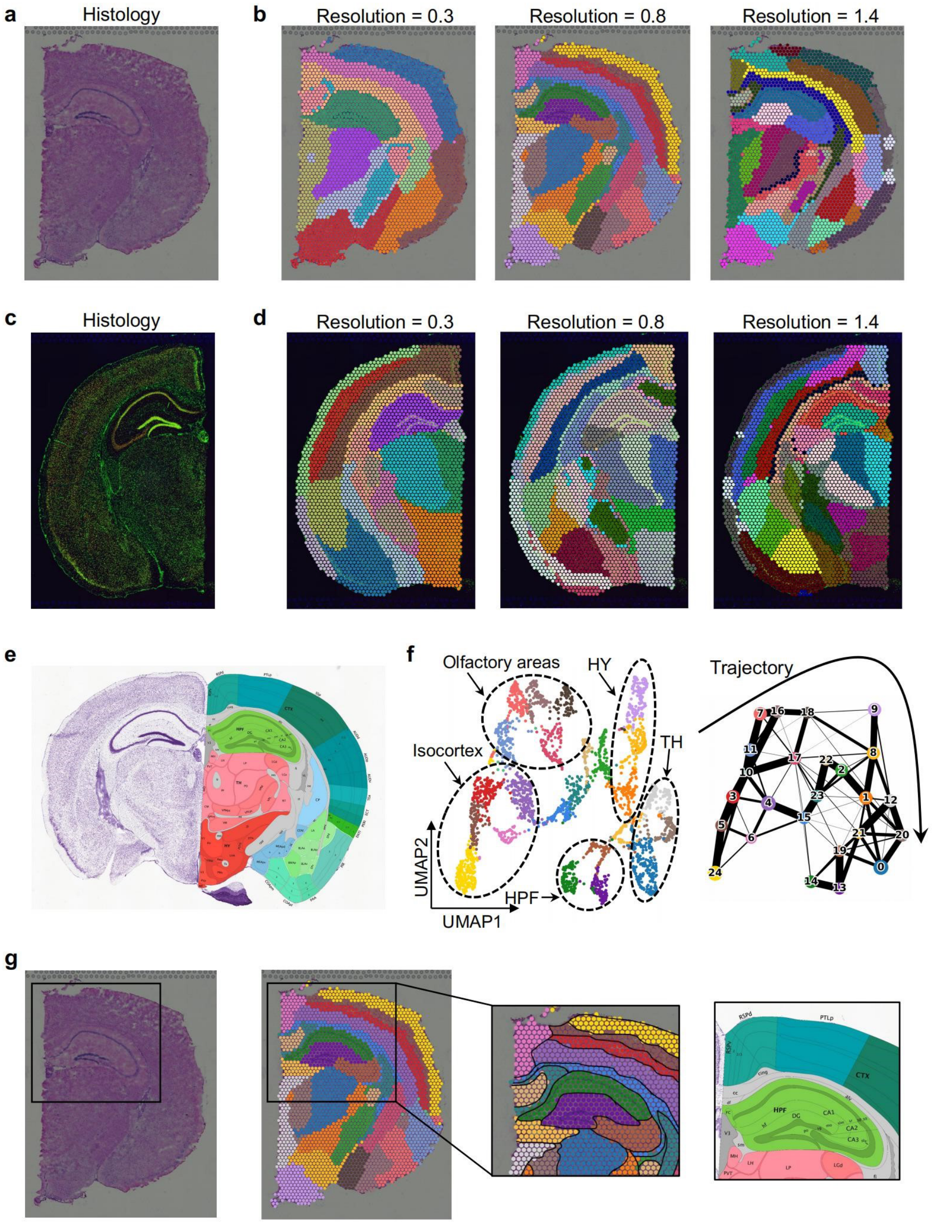

Supplementary Figure 14. Application of PROST on the mouse coronal cerebellum sequenced by Slide-seq. a, The annotation of the mouse cerebellum from the Allen Reference Atlas[22]. **b**, Manually assigned annotation[21]. **c**, Domain segmentation results from HMRF, SpaGCN, SpaceFlow, STAGATE, BASS, and PROST. Each box displays a zoomed-in view of the cerebellar cortex layers identified by various methods.

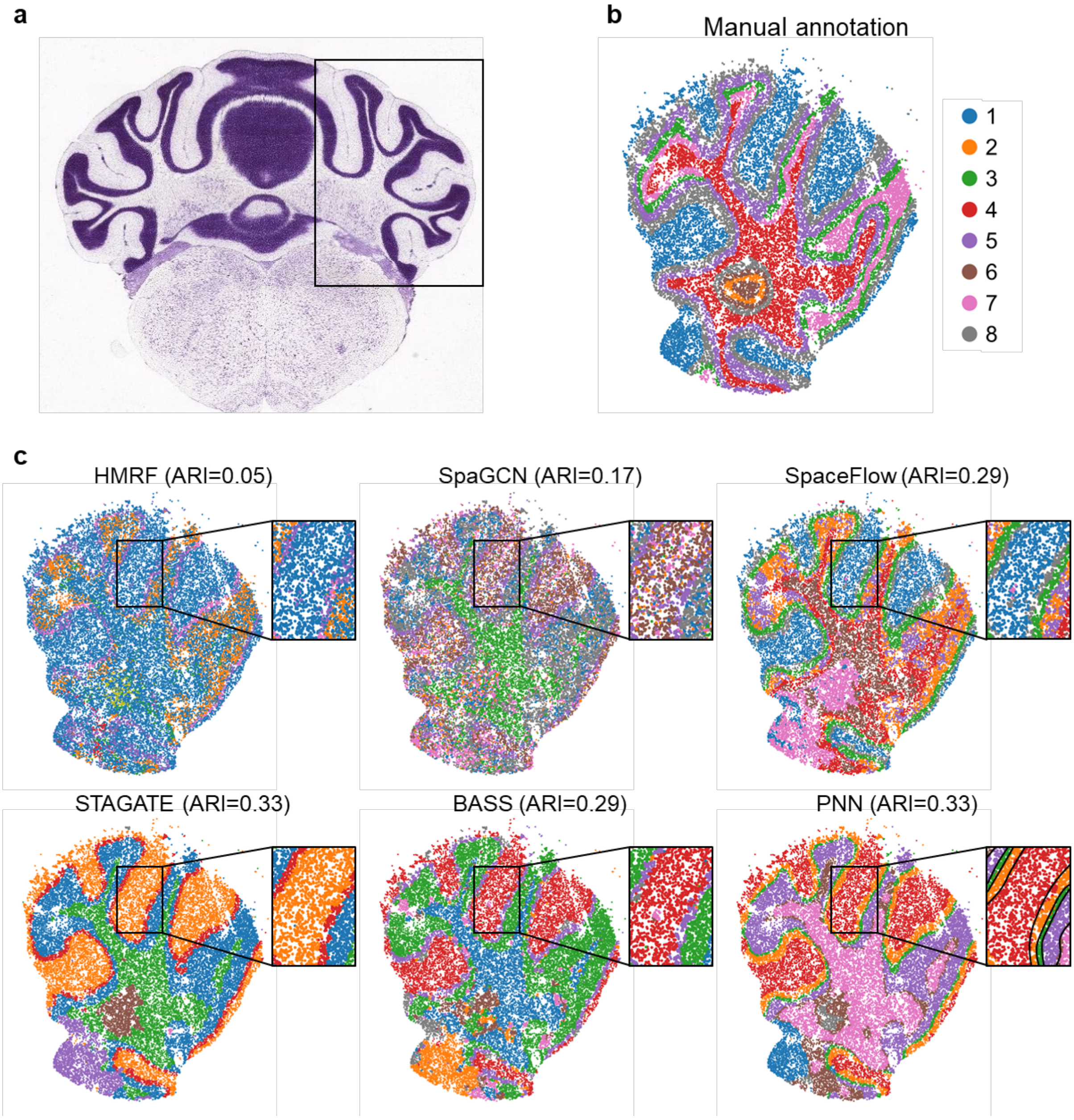

Supplementary Figure 15. PROST analysis on Slide-seq V2 mouse olfactory bulb dataset. a, The annotation from the Allen Reference Atlas of mouse olfactory bulb[22]. **b**, Domain segmentation produced by PROST, where Domain8 represents the spots outside the tissue section, which might be caused by RNA molecule's diffusion during tissue permeabilization in the experiment. **c**, UMAP visualization colored by the clusters of spots, using low-dimensional representation from PROST. **d**, Visualizations of spatial domains (up) identified by PROST and domain-specific marker genes (bottom).

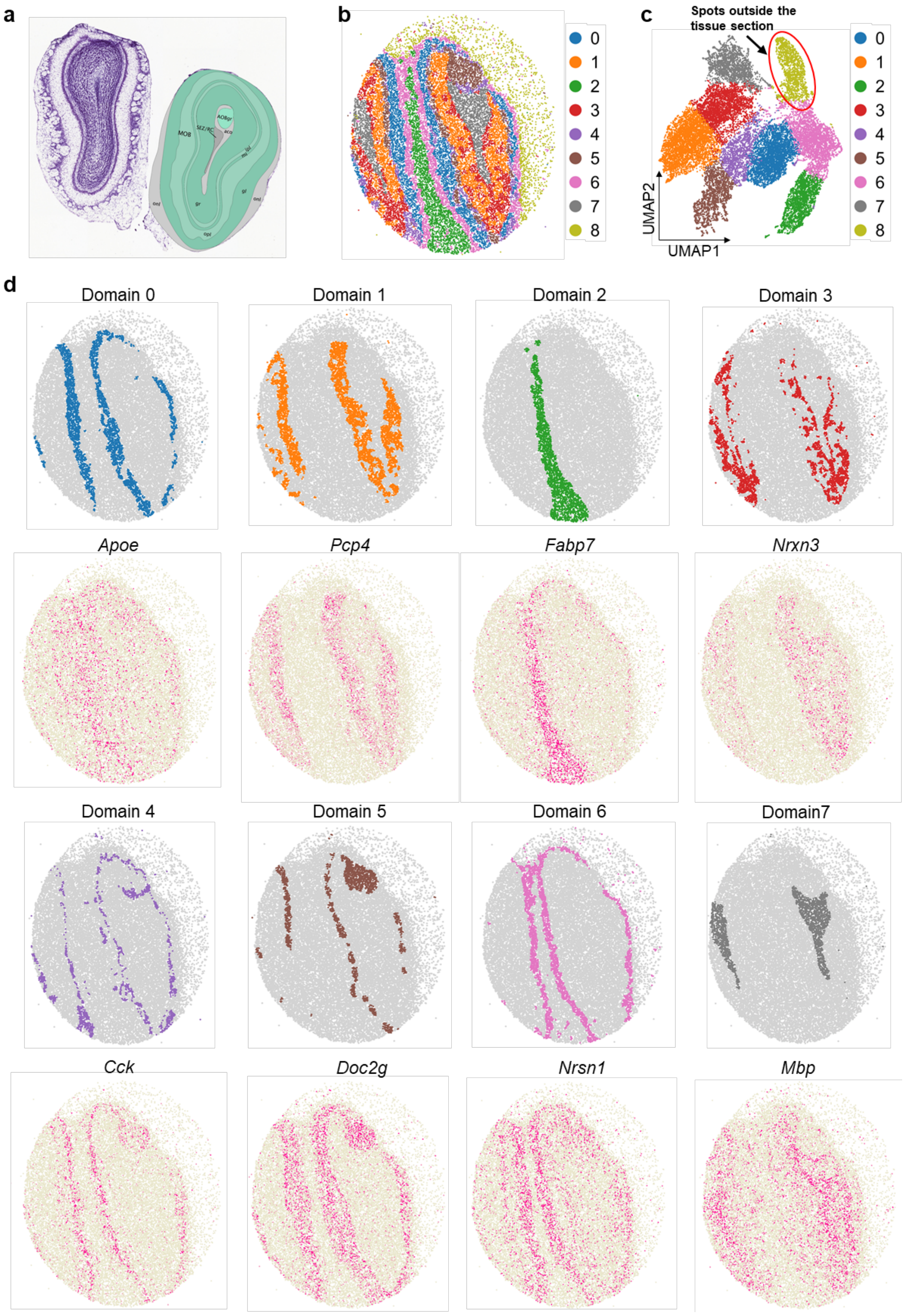

Supplementary Figure 16. Joint cluster analysis on the 10x Visium Mouse Brain Sagittal Anterior and Posterior. **a**, H&E image of the mouse brain sagittal anterior tissue section. **b**, Domain segmentation of the Mouse Brain Sagittal Anterior dataset using PROST with 0.3, 0.8 and 1.3 resolution settings. **c**, H&E image of the mouse brain sagittal posterior tissue section. **d**, Domain segmentation of the Mouse Brain Sagittal Posterior dataset using PROST with 0.2, 0.7, and 1.1 resolution settings. **e**, Joint alignment of spatial domains identified by PROST in the mouse brain sagittal anterior and posterior**.** **f**, The annotation of the mouse brain sagittal from the Allen Mouse Brain Atlas[22].

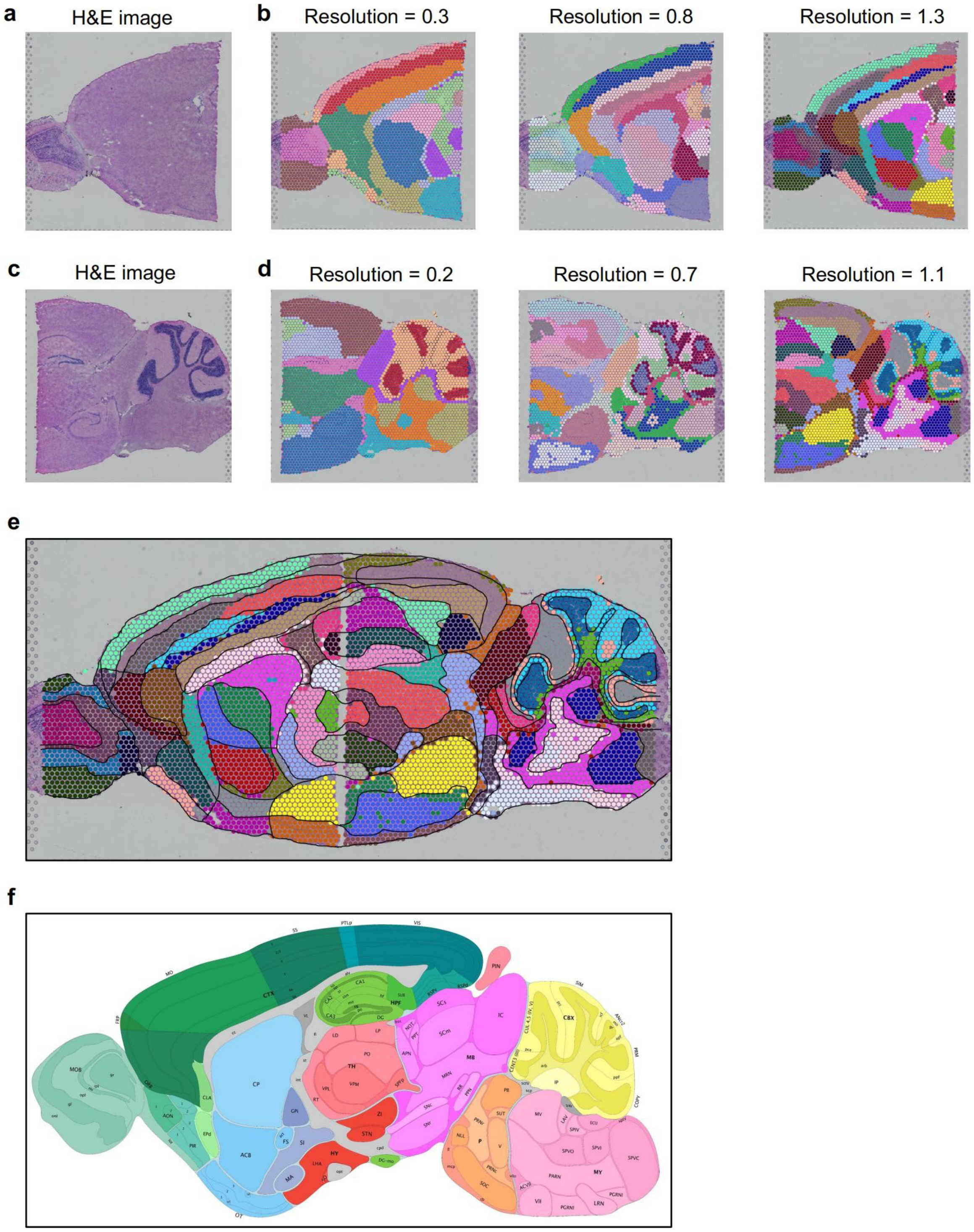

Supplementary Figure 17. **Spatial expression patterns for the top-ranked SVGs detected by PROST Index in the 10x Visium Mouse Brain Sagittal Anterior (a) and Mouse Brain Sagittal Posterior (b) datasets**. PI scores are shown in parentheses for each gene. Related to **Supplementary Figure 16**.

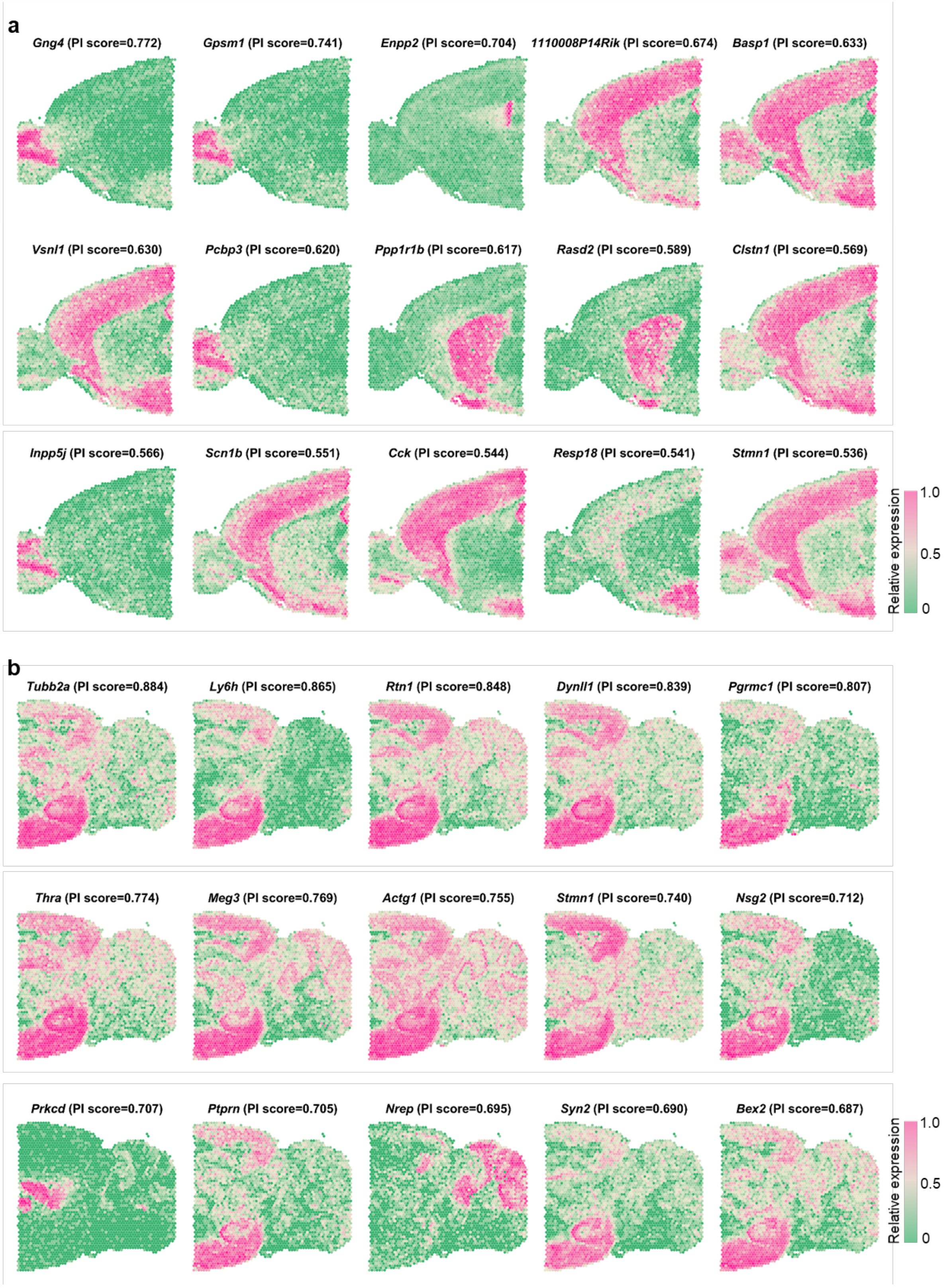

Supplementary Figure 18. Domain segmentation generated by PROST using 10x Visium Human Lymph Node dataset. a, H&E image of the human lymph node tissue section. **b**, Domain segmentation using PROST with 0.3, 0.7, 0.9, and 1.0 (default) resolution settings. **c**, UMAP visualizations are colored by the clusters of spots, using a low-dimensional representation from PROST with 0.3, 0.7, 0.9, and 1.0 resolution settings.

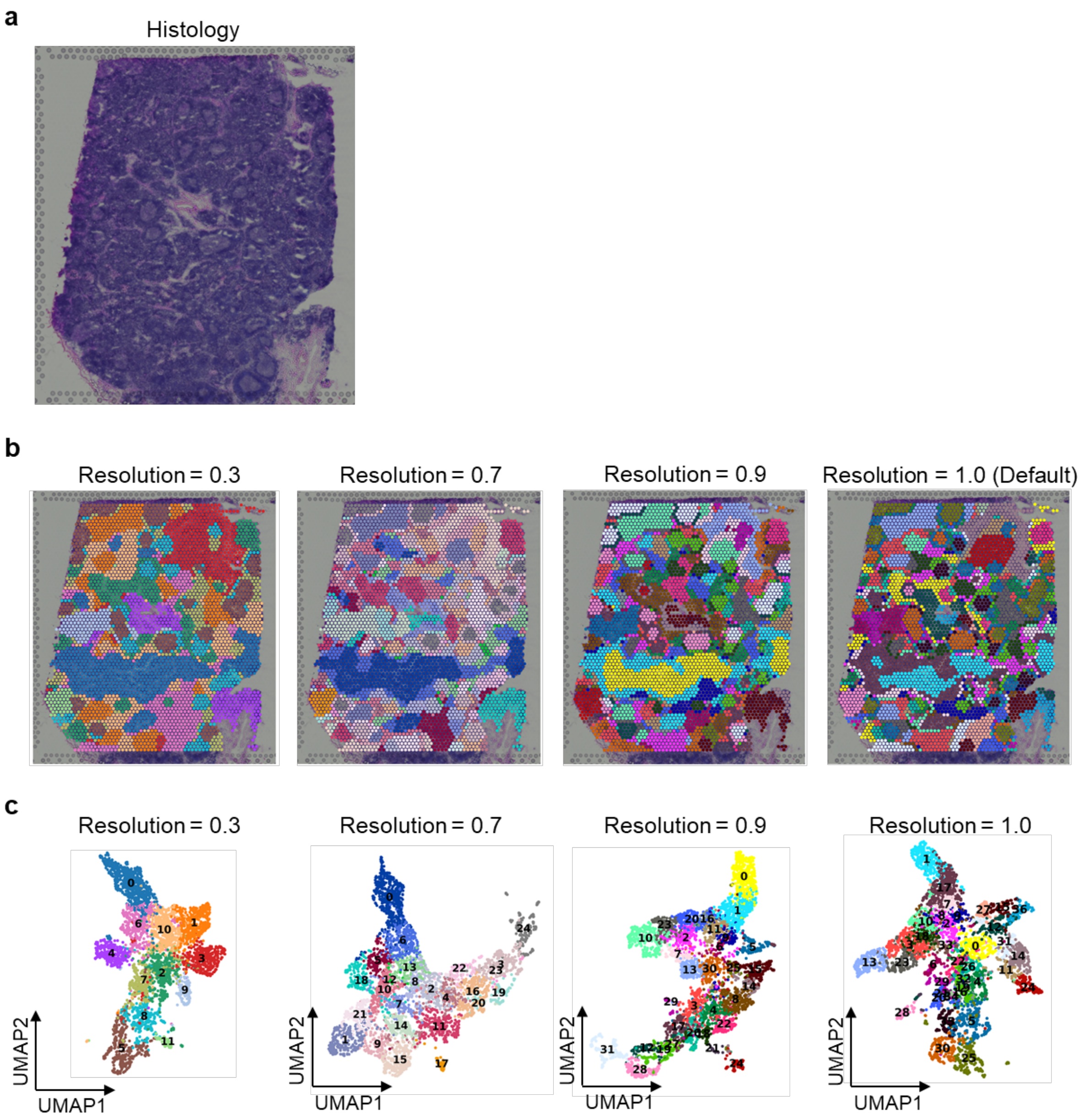

Supplementary Figure 19. Spatial expression patterns for the top-ranked SVGs detected by PROST Index in the 10x Visium Human Lymph Node dataset. PI scores are shown in parentheses for each gene. Related to **Supplementary Figure 18**.

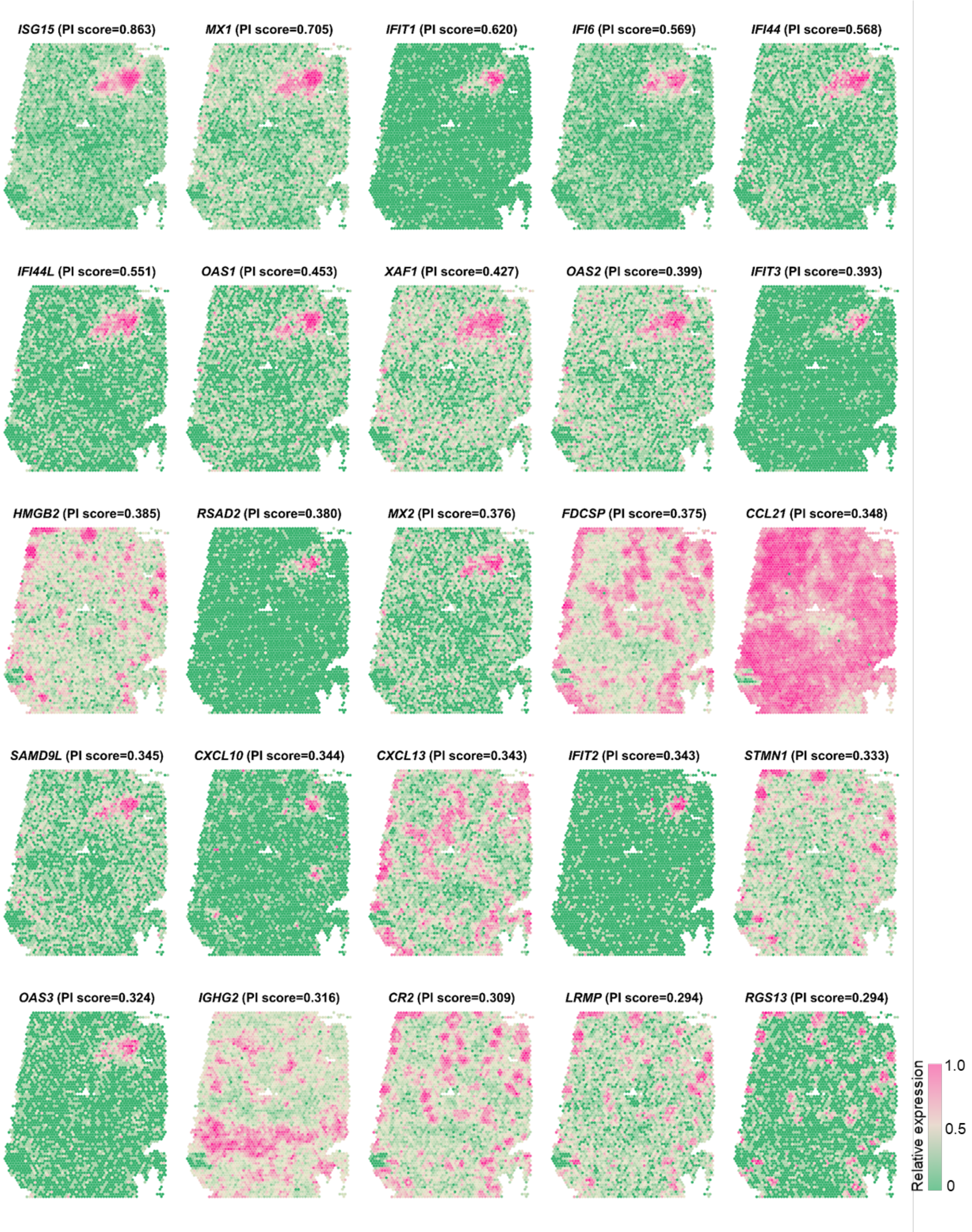

Supplementary Figure 20. Domain segmentation generated by PROST using the 10x Visium FFPE Mouse Kidney dataset. a, Histological image of the mouse kidney tissue section. **b**, Domain segmentation using PROST with 0.2, 0.7,1.0 (default) and 1.3 resolution settings. **c**, UMAP visualizations colored by the clusters of spots, using a low-dimensional representation from PROST with 0.2, 0.7, 1.0, and 1.3 resolution settings.

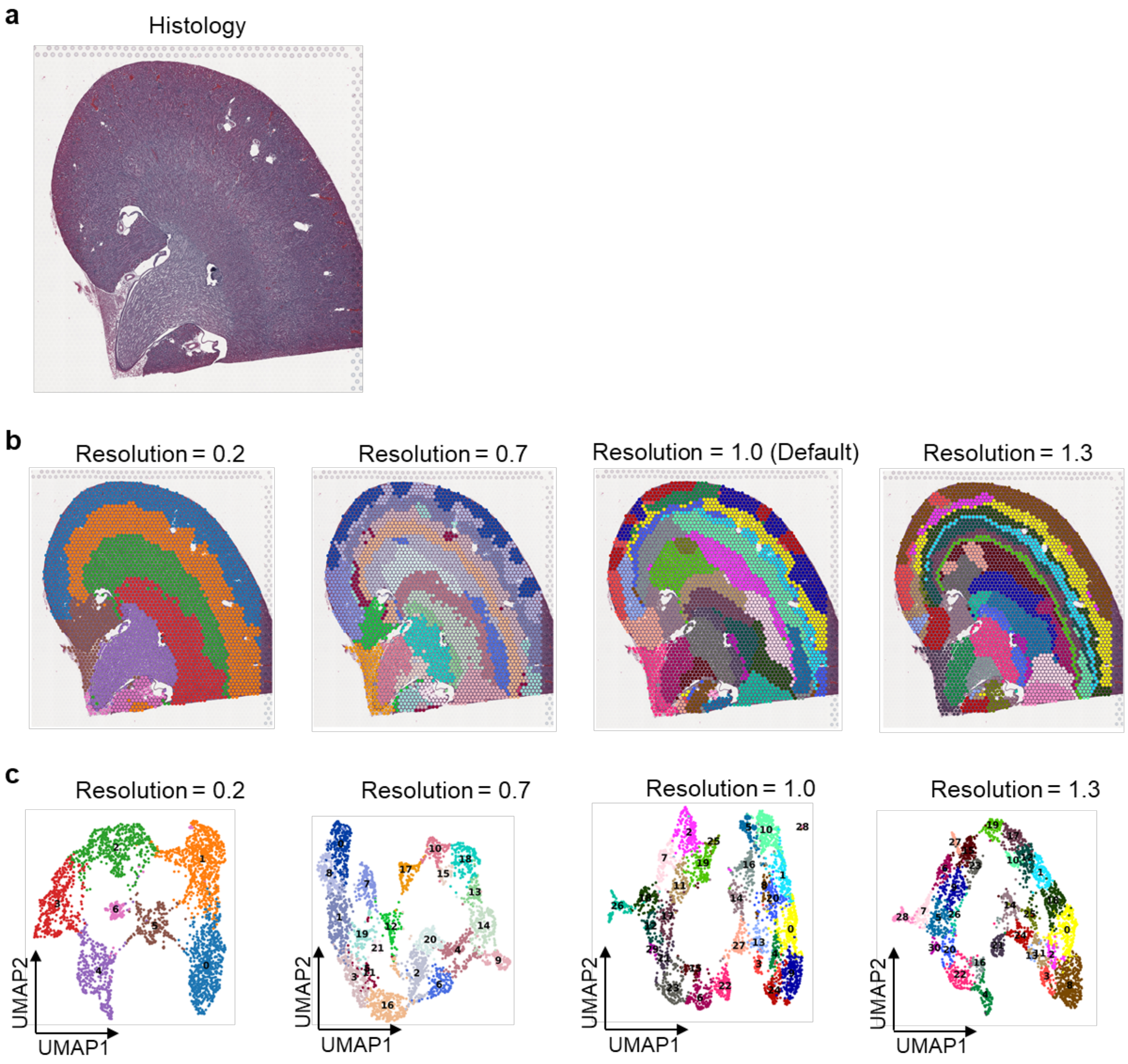

Supplementary Figure 21. Spatial expression patterns for the top-ranked SVGs detected by PROST Index in the 10x Visium FFPE Mouse Kidney dataset. PI scores are shown in parentheses for each gene. Related to **Supplementary Figure 20**.

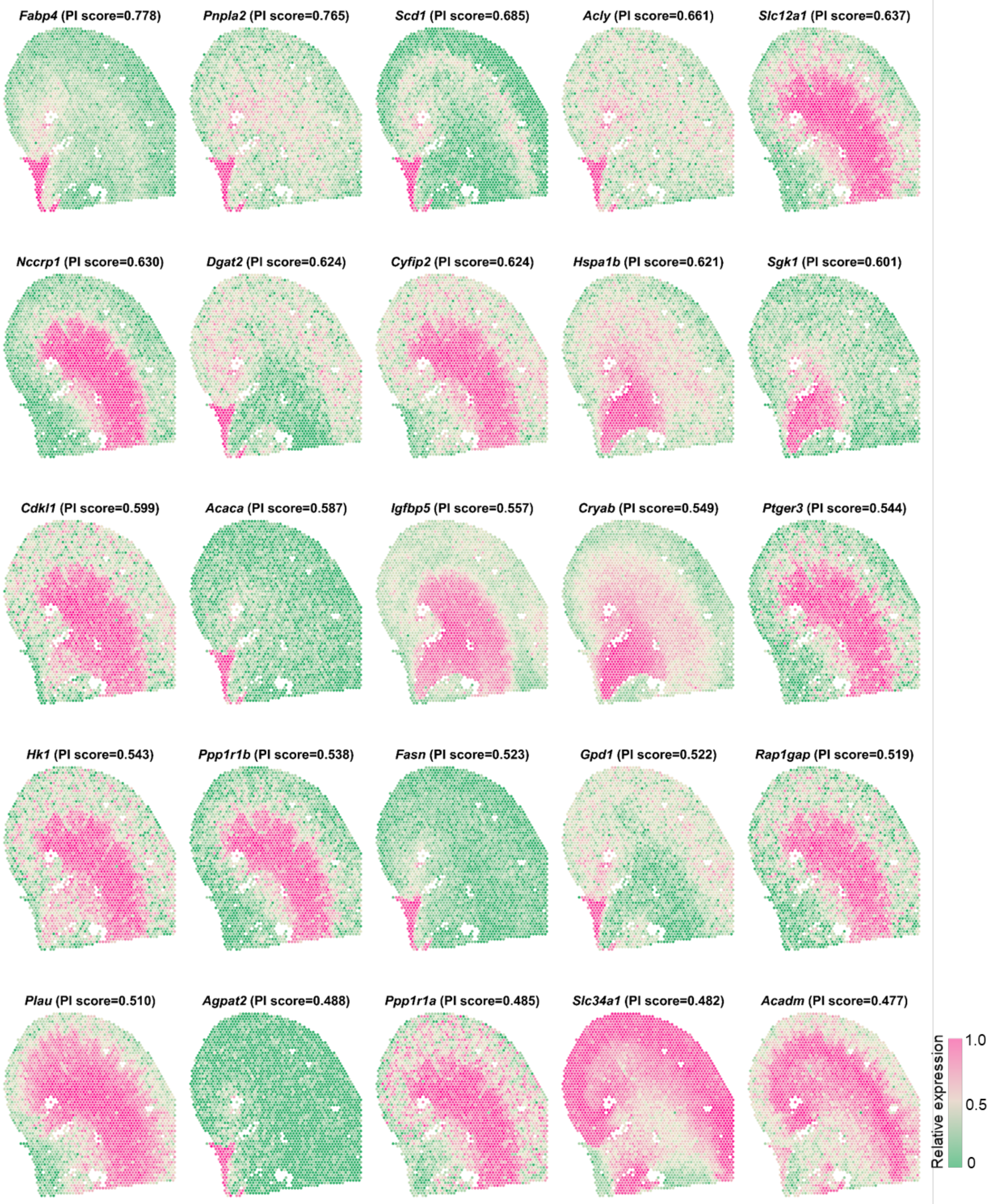

Supplementary Figure 22. Comparison of SVGs identified by four methods using the DLPFC dataset with section ID 151672. a, Venn diagram shows the intersections of SVGs identified by Seurat, SpatialDE, SPARK-X, SINFONIA and PROST, respectively. Source data are provided as a Source Data file. **b**, Venn diagram shows the intersection of the 50 top-ranked SVGs identified by Seurat, SpatialDE, SPARK-X, SINFONIA, and PROST, respectively. Source data are provided as a Source Data file. **c**, Venn diagram shows the intersection of the 50 top-ranked SVGs identified by SPARK-X and PROST. Source data are provided as a Source Data file. **d**, Boxplots shows Moran's *I* and Geary's *C* value for 10 SVGs that were only identified by SPARK-X ('SPARK-X only') or PROST ('PROST only'), respectively. Source data are provided as a Source Data file. **e**, Boxplots shows the PROST Index, *Significance,* and *Separability* value for 10 SVGs that were only identified by SPARK-X ('SPARK-X only') or PROST ('PROST only'), respectively. Source data are provided as a Source Data file. **f**, Spatial expression patterns for 10 SVGs exclusively identified by top 50 of SPARK-X. Q-values are shown in parentheses for each gene. **g**, Spatial expression patterns for 10 SVGs exclusively identified by top 50 of PROST. PI scores are shown in parentheses for each gene.

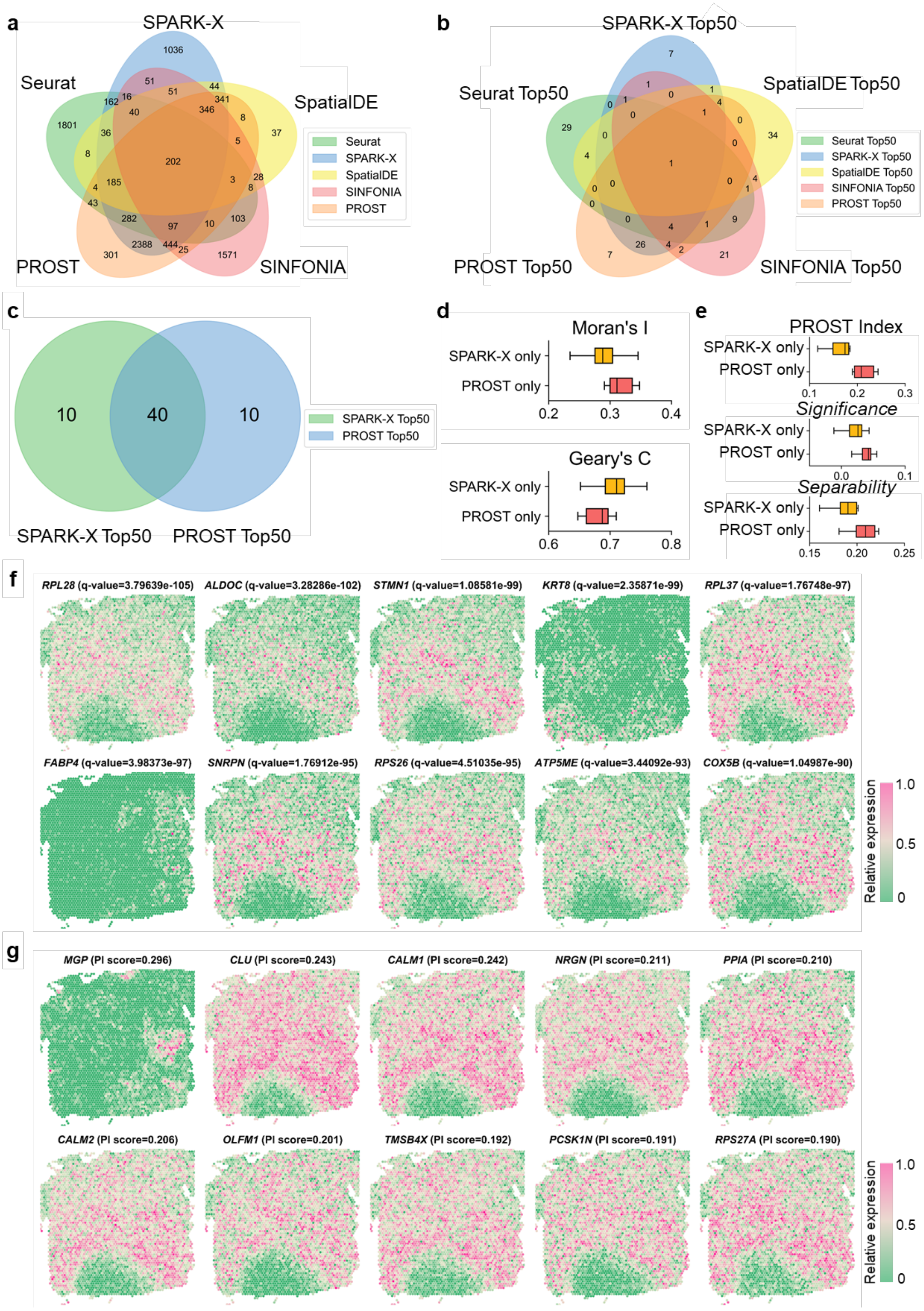
a
SPARK-X
Seurat
SpatialDE
PROST
SINFONIA
1036
51
162
16
51
44
341
40
346
8
1801
36
37
5
8
202
4
185
3
28
8
43
282
97
10
103
2388
444
25
301
1571
Seurat
SPARK-X
SpatialDE
SINFONIA
PROST
b
SPARK-X Top50
Seurat Top50
SpatialDE Top50
PROST Top50
SINFONIA Top50
7
Seurat Top50
SPARK-X Top50
SpatialDE Top50
SINFONIA Top50
PROST Top50
29
34
26
7
21
c
10
40
10
SPARK-X Top50
PROST Top50
SPARK-X Top50
PROST Top50
d
Moran's I
SPARK-X only
PROST only
0.2
0.3
0.4
Geary's C
SPARK-X only
PROST only
0.6
0.7
0.8
e
PROST Index
SPARK-X only
PROST only
0.1
0.2
0.3
Significance
SPARK-X only
PROST only
0.0
0.1
Separability
SPARK-X only
PROST only
0.15
0.20
0.25
f
RPL28 (q-value=3.79639e-105)
ALDOC (q-value=3.28286e-102)
STMN1 (q-value=1.08581e-99)
KRT8 (q-value=2.35871e-99)
RPL37 (q-value=1.76748e-97)
FABP4 (q-value=3.98373e-97)
SNRPN (q-value=1.76912e-95)
RPS26 (q-value=4.51035e-95)
ATP5ME (q-value=3.44092e-93)
COX5B (q-value=1.04987e-90)
Relative expression
1.0
0.5
0
g
MGP (PI score=0.296)
CLU (PI score=0.243)
CALM1 (PI score=0.242)
NRGN (PI score=0.211)
PPIA (PI score=0.210)
CALM2 (PI score=0.206)
OLFM1 (PI score=0.201)
TMSB4X (PI score=0.192)
PCSK1N (PI score=0.191)
RPS27A (PI score=0.190)
Relative expression
1.0
0.5
0

Supplementary Figure 23. Transportability of SVGs across DLPFC sections. Spatial expression patterns for representative SVGs, i.e. *MBP* (**a**), *TMSB10* (**b**), *SAA1* (**c**), *PCP4* (**d**), *MGP* (**e**) and *CALM1* (**f**) across 12 sections from 10x Visium DLPFC dataset, showing transportability of SVGs across sections.

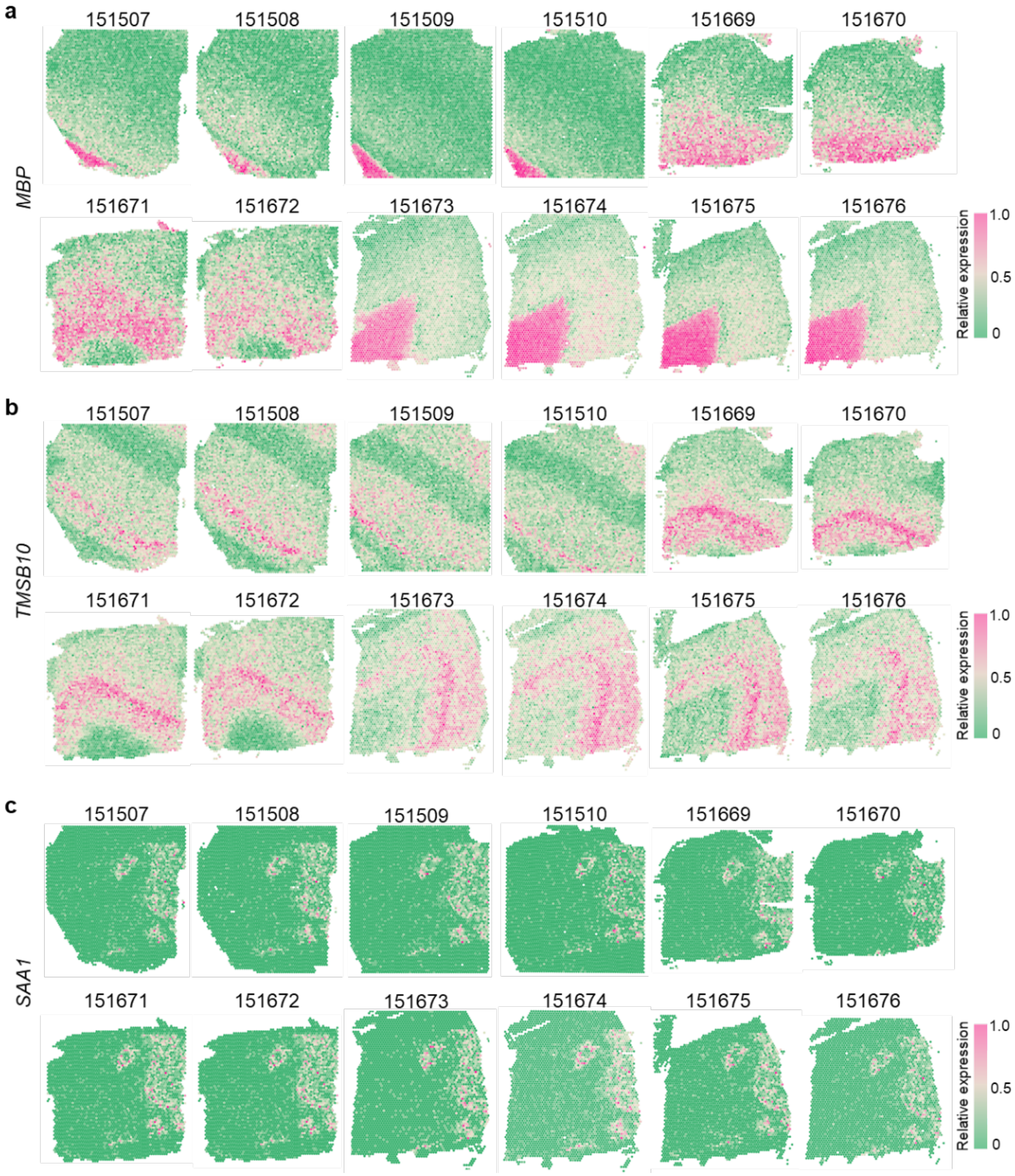

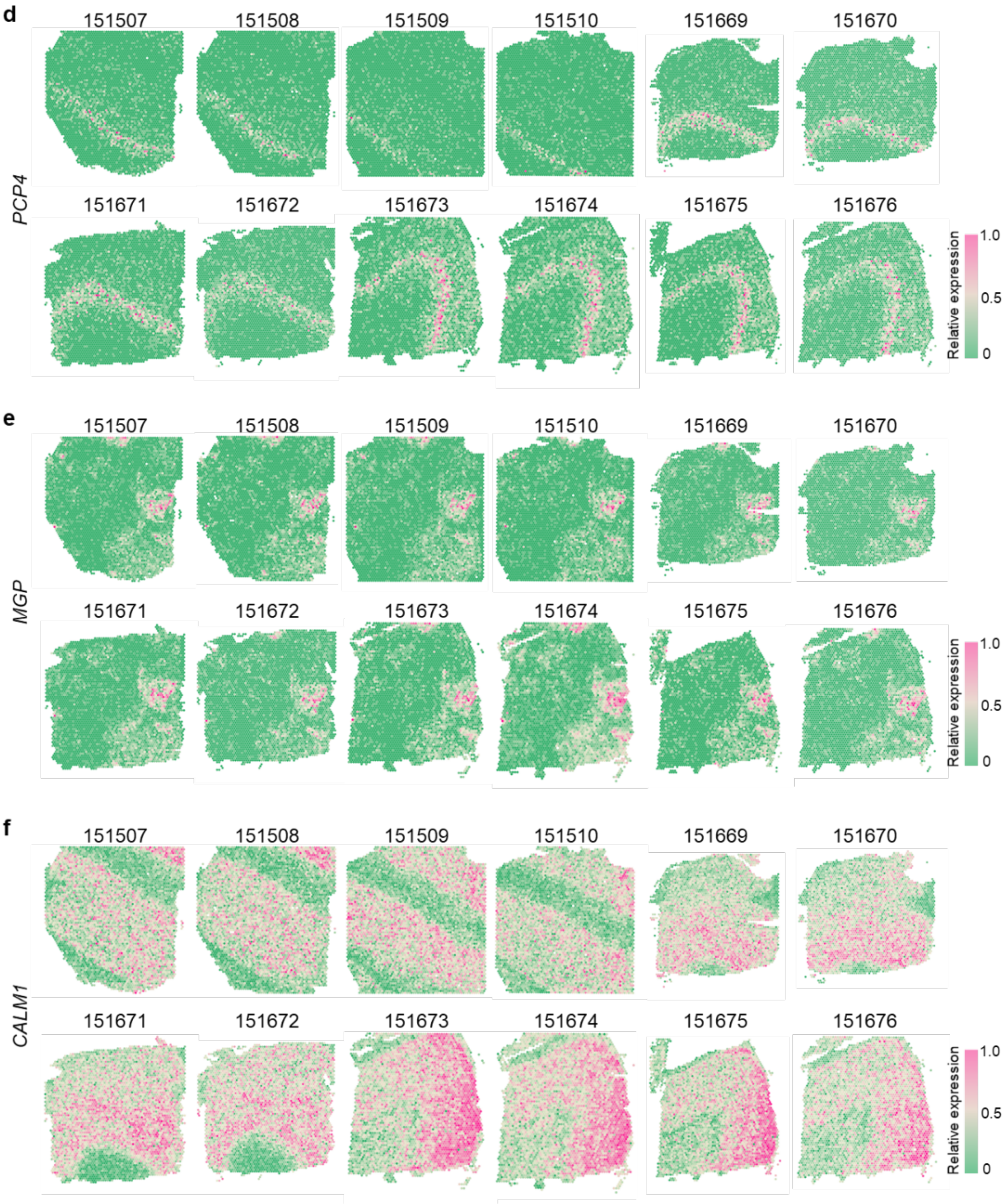

d
PCP4
151507
151508
151509
151510
151669
151670
151671
151672
151673
151674
151675
151676
Relative expression
1.0
0.5
0
e
MGP
151507
151508
151509
151510
151669
151670
151671
151672
151673
151674
151675
151676
Relative expression
1.0
0.5
0
f
CALM1
151507
151508
151509
151510
151669
151670
151671
151672
151673
151674
151675
151676
Relative expression
1.0
0.5
0

Supplementary Figure 24. PI-based SVG identification. Venn diagrams show the intersections between SVGs with PI scores greater than 0 and those with FDR values under 0.05, considering parametric test, non-parametric test, and the combination of both parametric and non-parametric tests. The datasets under analysis encompass a diverse range of tissues and experimental conditions.

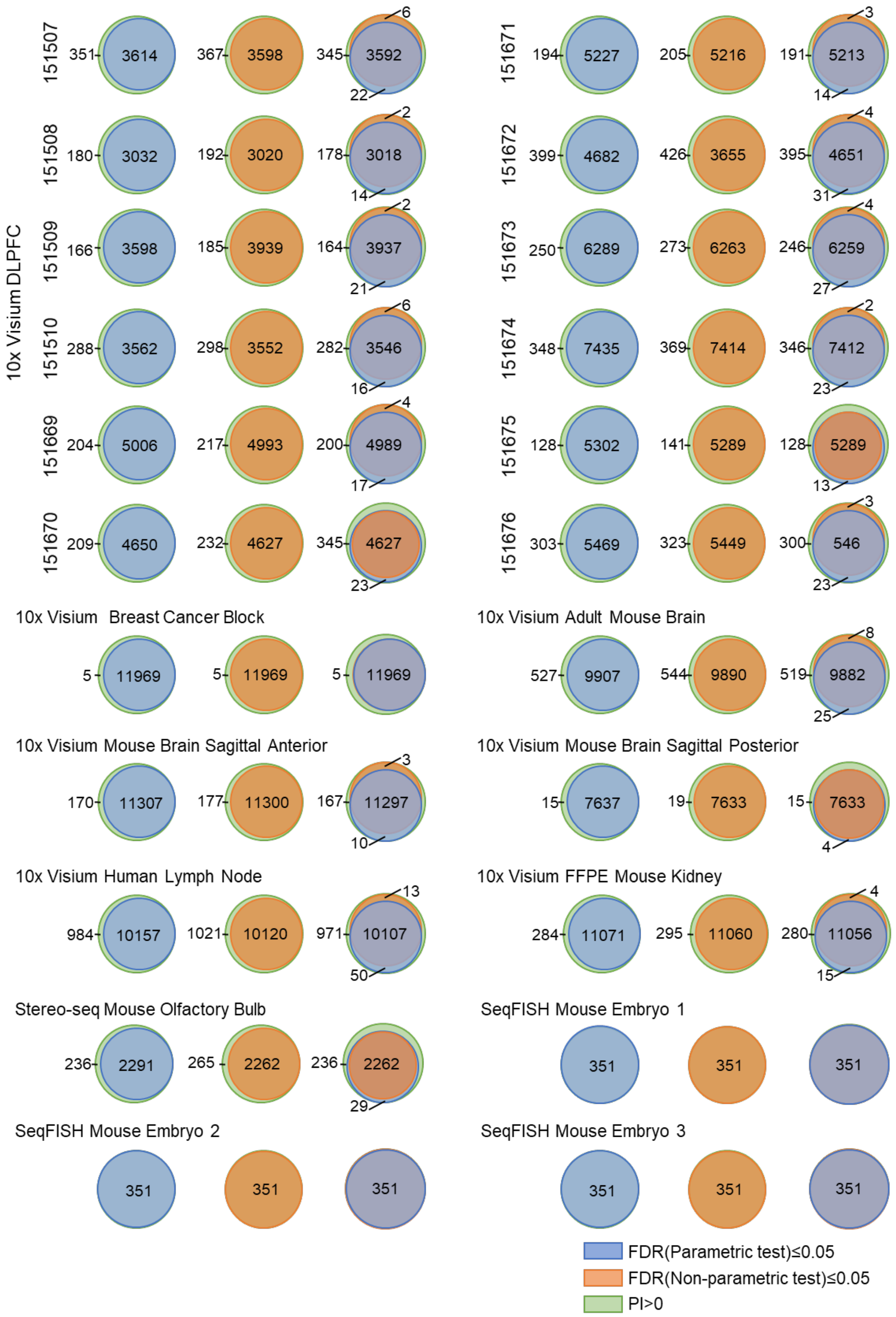

Supplementary Figure 25. Comparative analysis of SVG identification performances between PI and scGCO based on the simulated dataset[12] under various noisy conditions. a, Line plots show the accuracy, sensitivity, false positive rate, and F1 score in the SVG identification performance for PI and scGCO under the noisy conditions with an increase in Gaussian noise levels. For each simulation scenario, the values of true negatives (TN), true positives (TP), false negatives (FN), and false positives (FP) were calculated. These values were then used to compute the following four metrics: Accuracy = (TP + TN)/(TP + TN + FP + FN); Sensitivity = TP/(TP + FN); False positive rate (FPR) = FP/(FP + TN), and F1 score (F1) = 2∗TP/(2∗TP + FN + FP). Source data are provided as a Source Data file. **b,** The top row displays synthetic expression patterns of typical spatial patterns (Pattern 1 to 3) under different noisy conditions as Gaussian noise levels increase. The subsequent row illustrates the corresponding changes in FDR in identifying the synthetic patterns as SVGs under different noisy conditions. Source data are provided as a Source Data file.

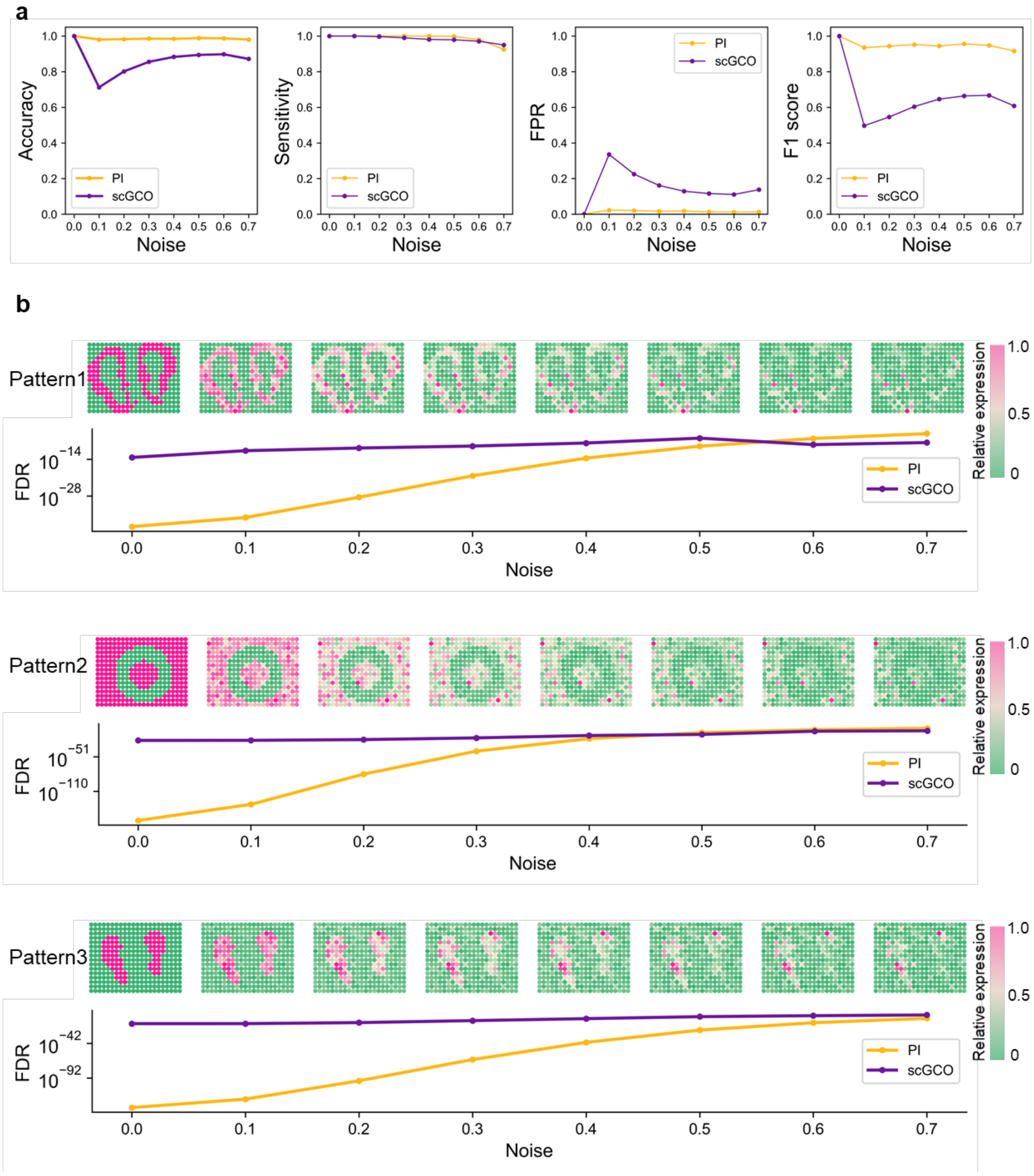

Supplementary Figure 26. Comparative analysis of SVG identification performances between NN-based method STAGATE and PI, using simulated mouse somatosensory cortex dataset[21] under various noisy conditions. a, Line plots show the accuracy, sensitivity, false positive rate, and F1 score in the SVG identification performance for STAGATE and PI under the noisy conditions with an increase in Gaussian noise levels. For each simulation scenario, the values of true negatives (TN), true positives (TP), false negatives (FN), and false positives (FP) were calculated, as described in **Supplementary Figure 25**. Source data are provided as a Source Data file. **b,** The top row displays the expression patterns of representative SVGs under different noisy conditions as Gaussian noise levels increase. The subsequent row illustrates the corresponding changes in FDR in identifying the expression patterns as SVGs under different noisy conditions. Source data are provided as a Source Data file.

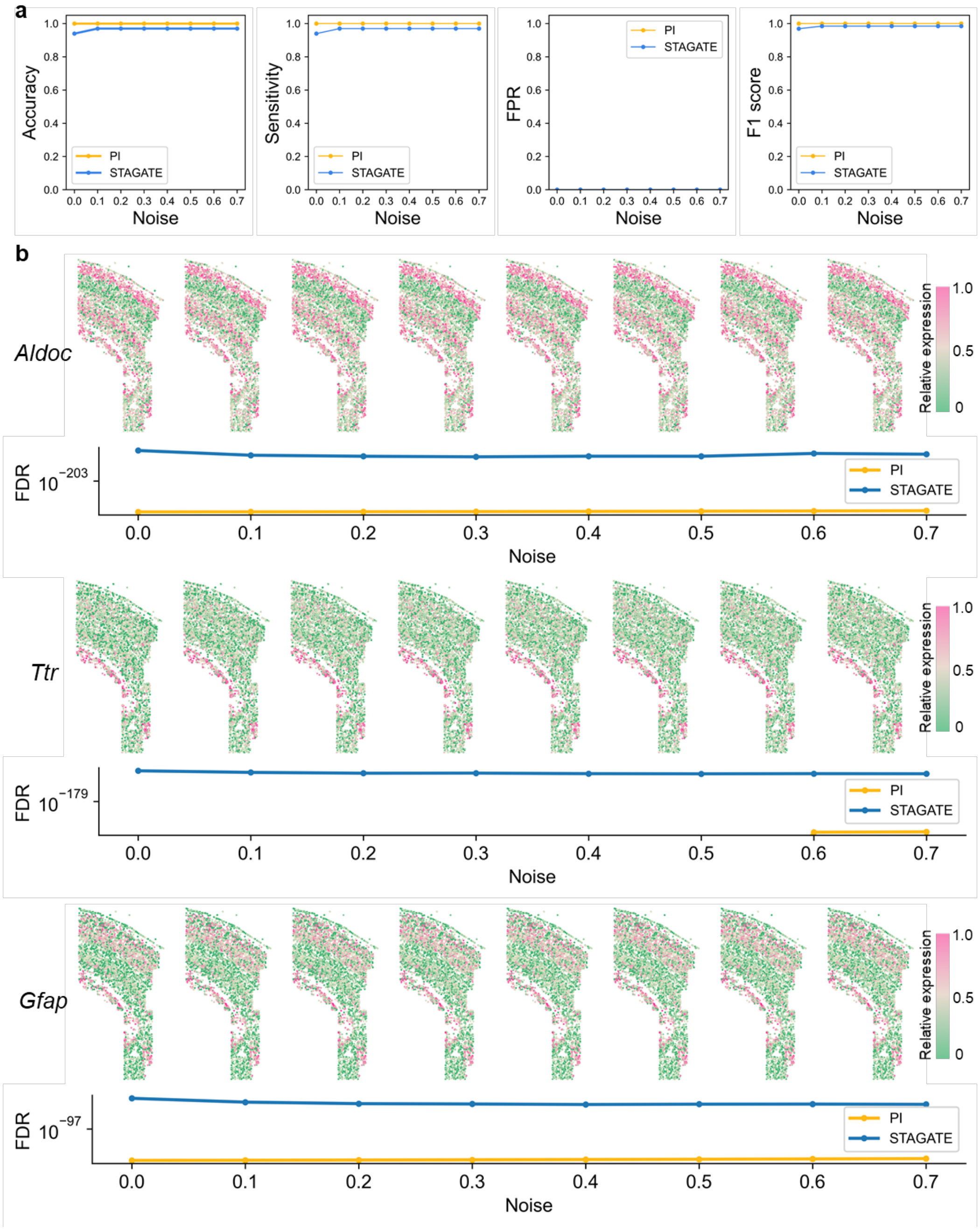

Supplementary Figure 27. Ablation experiments illustrating the influence of various SVG inputs on domain segmentation performance in PNN (a), SpaceFlow (b), STAGATE (c), and SpaGCN (d) using the 10x Visium DLPFC dataset. The x-axis denotes the different feature selection methods; "None" represents no feature selection, while the numbers following underscores indicate the quantity of features chosen by each respective method. Source data are provided as a Source Data file.

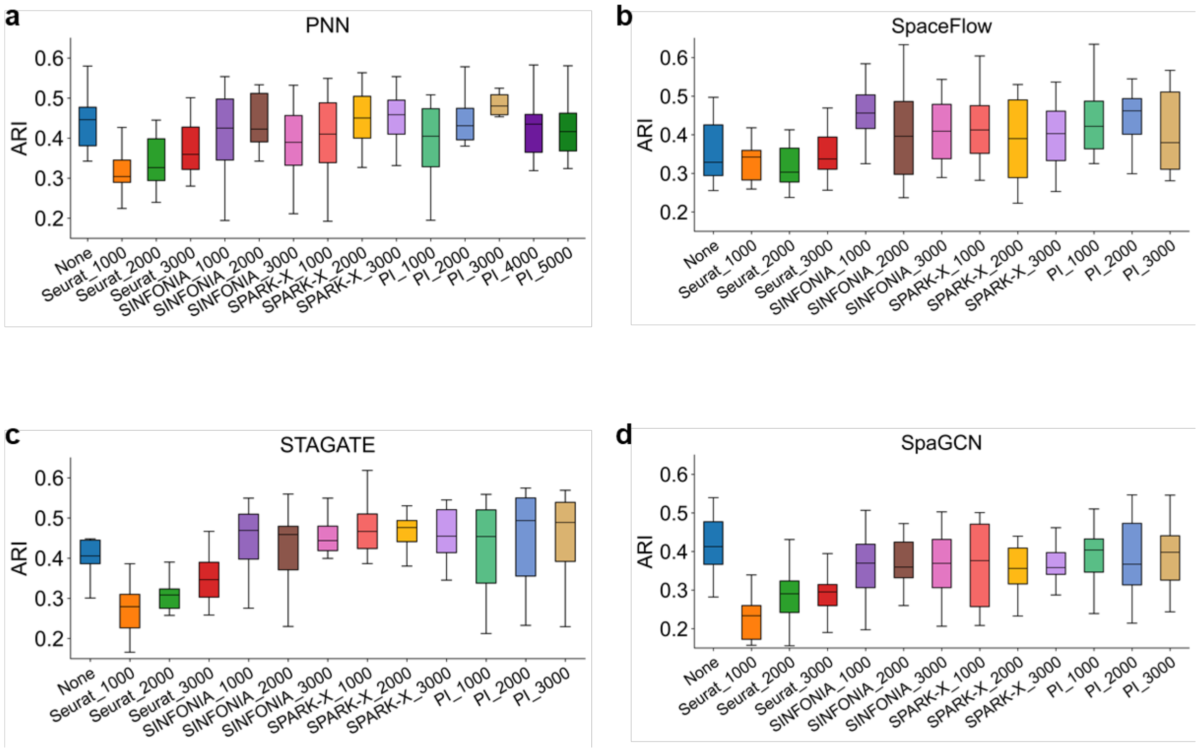

Supplementary Figure 28. Application of PROST on the mouse somatosensory cortex sequenced by osmFISH in different simulation scenarios. a, The annotation of the mouse somatosensory cortex from the Allen Reference Atlas[22]. **b**, Manual annotation of the real count data from the original study[17]. **c**, Domain segmentation generated by SpaGCN, SpaceFlow, STAGATE, BASS, and PROST. **d**, Spatial expression patterns of the gene *Rorb* at different dropout rates. **e**, The Line chart shows the performance of several methods at different dropout rates. Source data are provided as a Source Data file. **f**, Manual annotation of the simulated count data[21], followed by spatial domains identified by various methods.

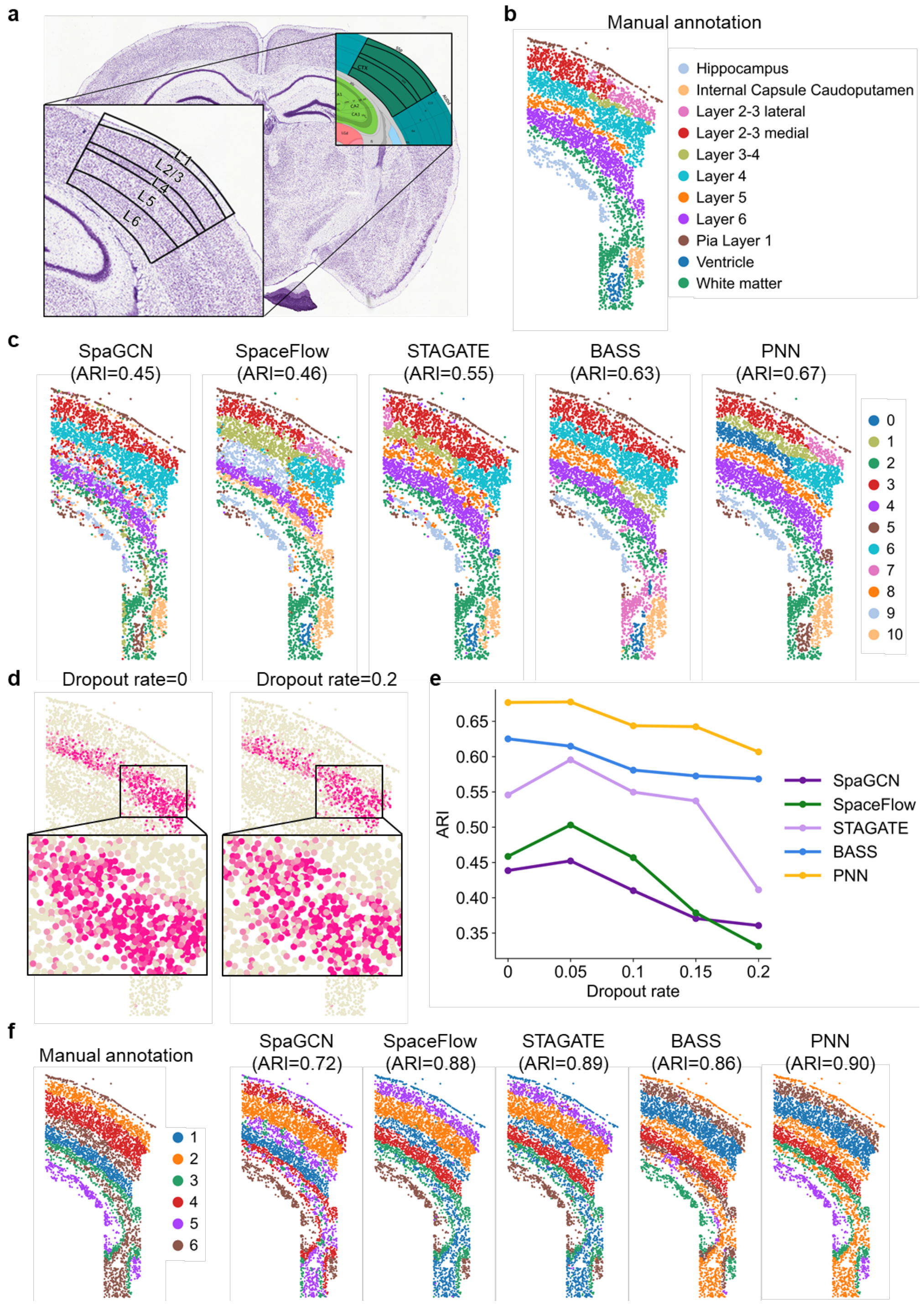

Supplementary Figure 29. Comparison of spatial domains from inside to outside in the Stereo-seq mouse olfactory bulb dataset. Spatial domains were segmented by PROST, BASS, SpaceFlow, STAGATE, and SpaGCN respectively.

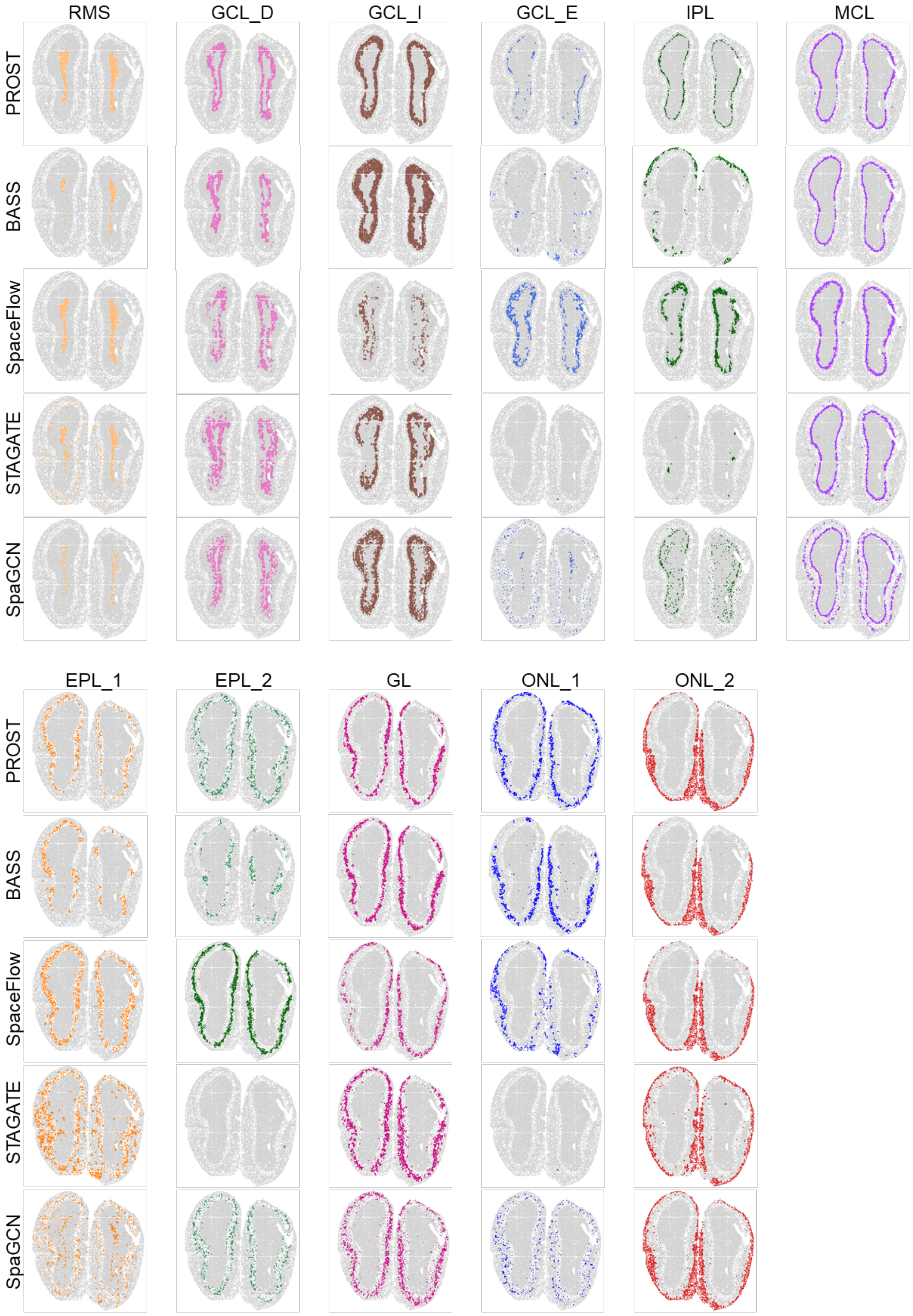

Supplementary Figure 30. PROST analysis on the Stereo-seq mouse olfactory bulb dataset. a UMAP visualizations colored by the annotation and marker gene of spots, using a low-dimensional representation from PROST in the Stereo-seq mouse olfactory bulb dataset. **b** Dot plot of the expression levels of domain-specific marker genes. The dot size represents the fraction of cells in a domain expressing the marker genes, and the color intensity represents the average expression levels of the marker gene in that domain. **c** Spatial expression patterns for the top-ranked SVGs detected by PI. PI scores are shown in parentheses for each gene.

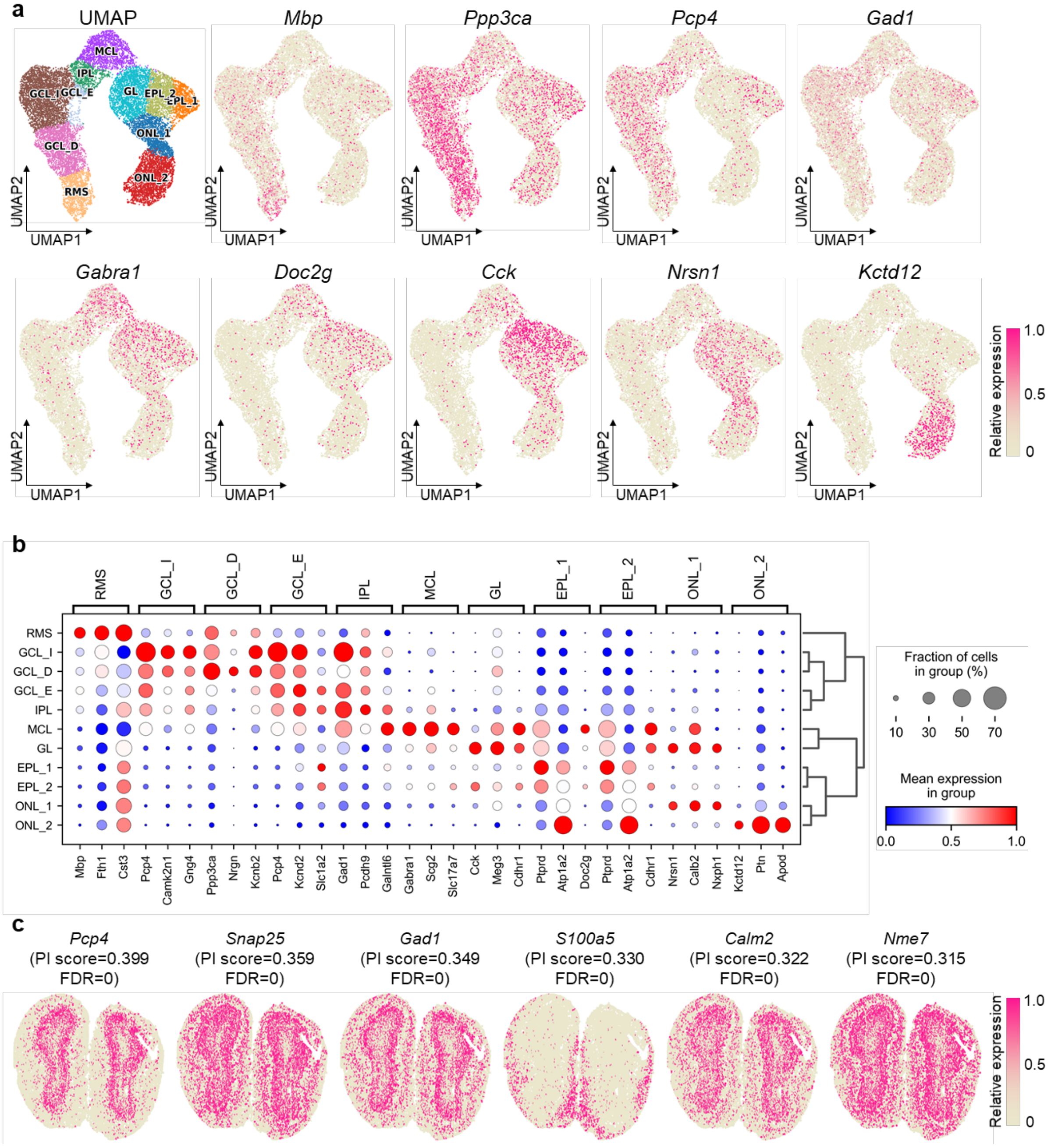

Supplementary Figure 31. Dot plot shows the expression levels of domain-specific marker genes on the SeqFISH mouse embryo dataset (Embryo 1). The dot size represents the fraction of cells in a domain expressing the marker gene, and the color intensity denotes the average expression level of the marker gene in a domain.

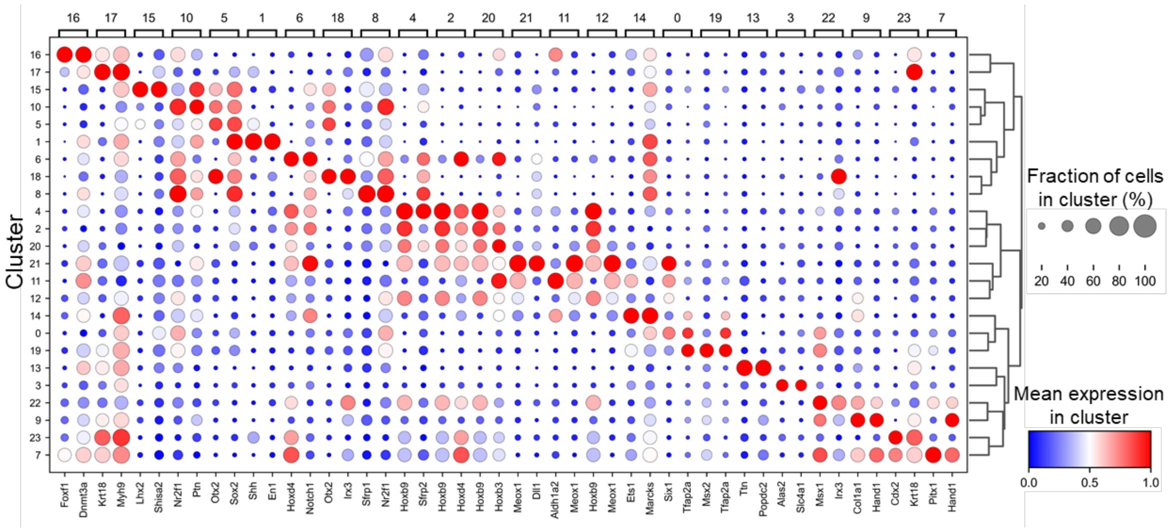

Supplementary Figure 32. PROST analysis on the SeqFISH mouse embryo dataset. a-b Annotation[20] and PROST segmentation of SeqFISH mouse embryo 2 (**a**) and embryo3 (**b**). **c-d** Spatial location of forebrain/midbrain/hindbrain (Embryo2) in annotation[20] (left of **c**) and the domains segmented by PROST (right of **c**), followed by the spatial expression of the corresponding marker genes (**d**). **e-f** Spatial location of forebrain/midbrain/hindbrain (Embryo3) in annotation[20] (left of **e**) and the domains segmented by PROST (right of **e**), followed by the spatial expression of the corresponding marker genes (**f**).

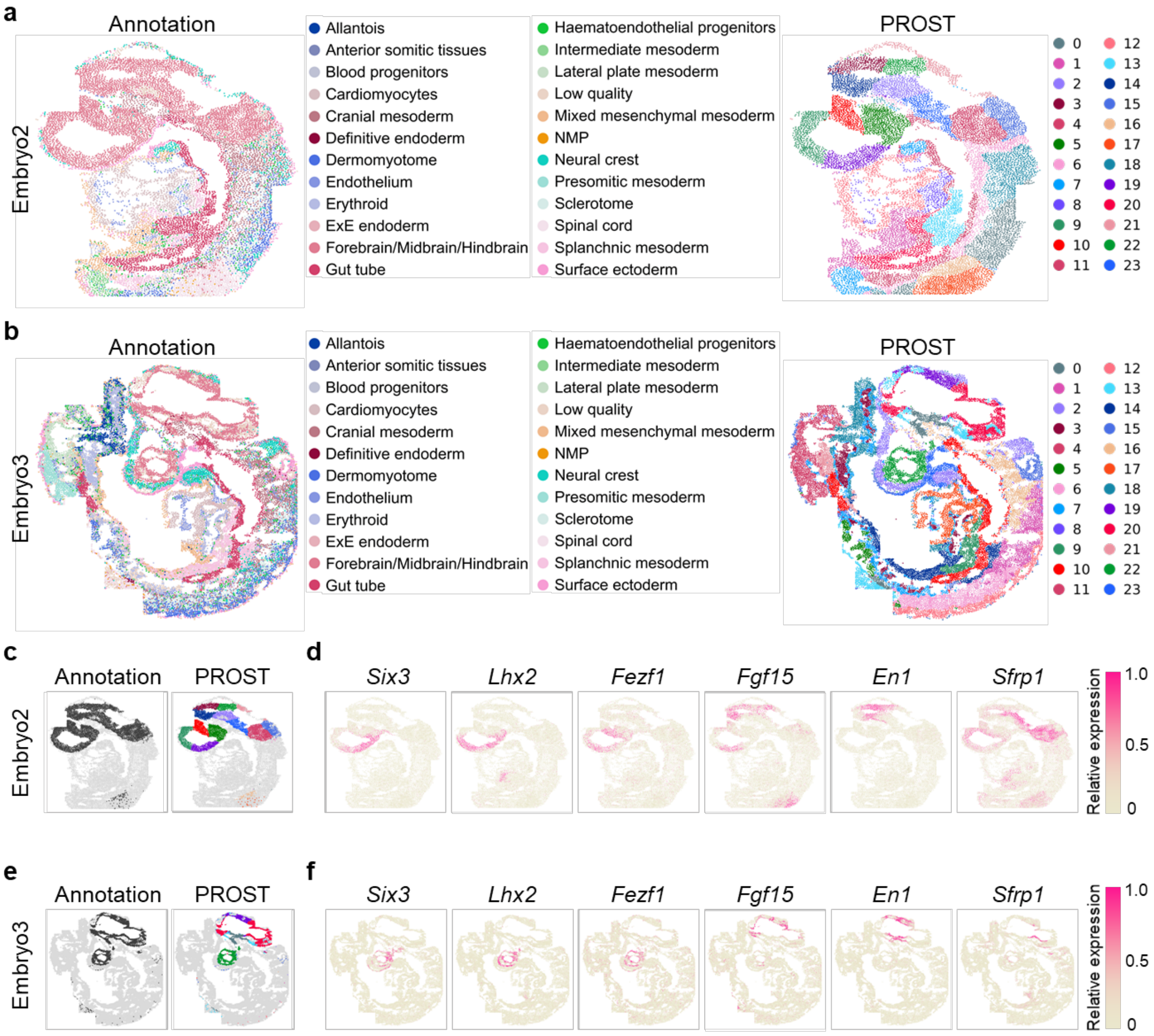

Supplementary Figure 33. 3D contour visualization illustrating PNN's sensitivity to parameters. The *k* neighbours, minimum distance, and max epoch parameters evaluated on the 10x Visium DLPFC (section 151672) dataset (**a**) and the osmFISH mouse somatosensory cortex dataset (**b**). As shown in **a**, the ARI consistently fluctuates between approximately 0.50 to 0.60 when the *k*-neighbor setting is adjusted from 4 to 12 and the Max epoch parameter ranges from 200 to 800 for the low-resolution 10x Visium dataset. Similarly, as illustrated in **b**, the ARI varies within a tight range of approximately 0.60 to 0.68 when the minimum distance setting is adjusted from 600 to 1,000 and the Max epoch parameter ranges from 100 to 1,000 for the high-resolution osmFISH dataset.

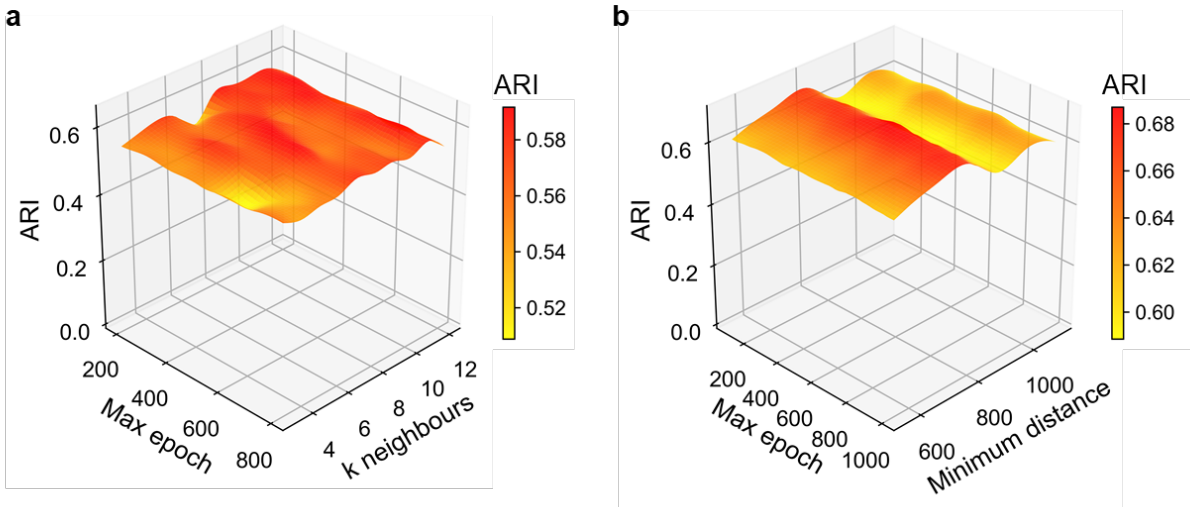

Supplementary Figure 34. Comparative assessments of the computational efficiency in terms of runtime and memory usage across various methods. The mouse cerebellum Slide-seq dataset that contains 25,551 spots with 17,729 genes was used. **a**, Efficiency comparison for SVG detection between PI and Seurat, SpatialDE, SPARK-X, and SINFONIA. Source data are provided as a Source Data file. **b**, Efficiency comparison for spatial domain identification between PNN and SpaGCN, BASS, SpaceFlow, and STAGATE. Source data are provided as a Source Data file.

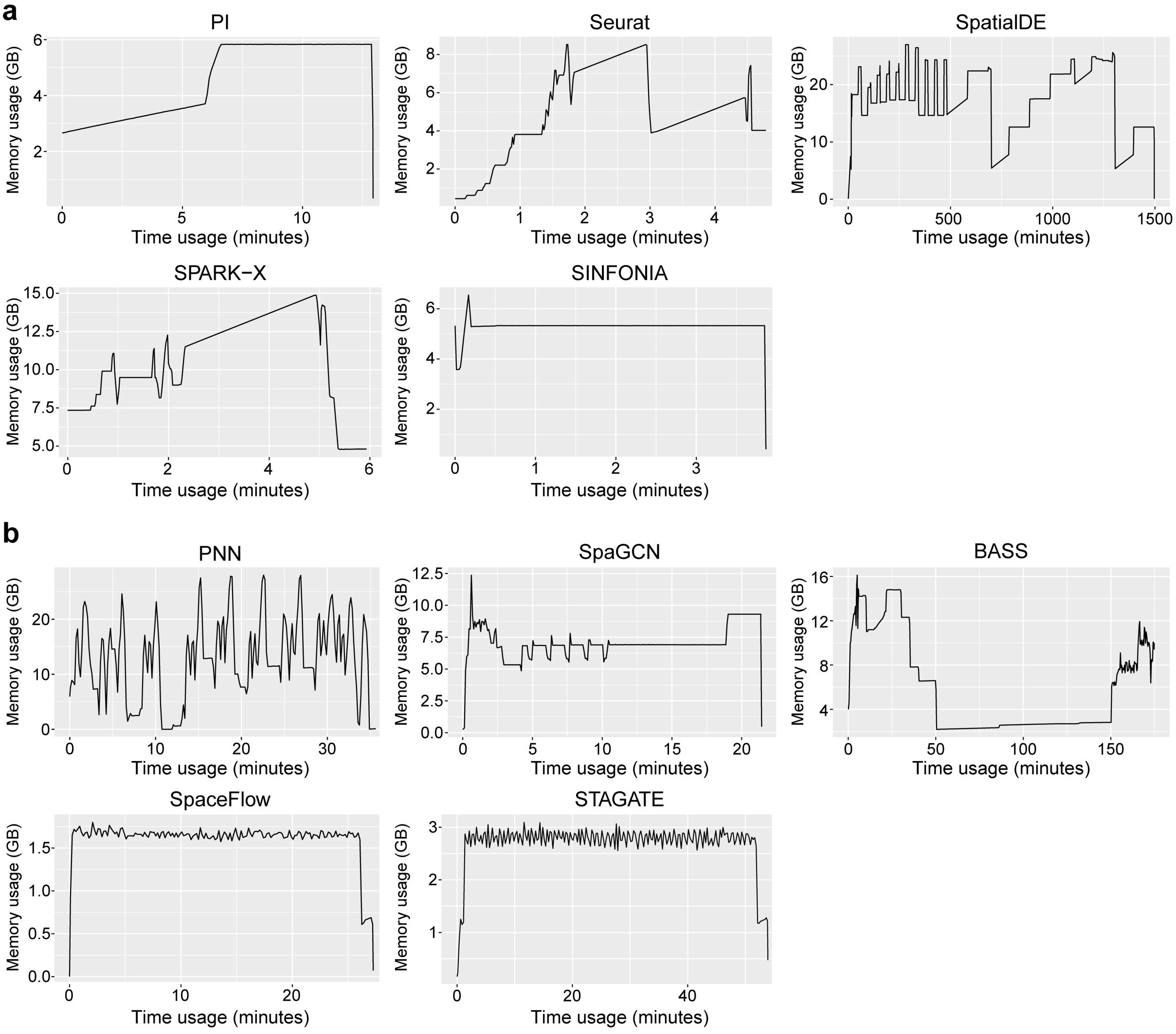

Supplementary Figure 35. Comparative assessments of the computational efficiency in terms of runtime and CPU consumption across various methods. The mouse cerebellum Slide-seq dataset that contains 25,551 spots with 17,729 genes was used. **a**, Efficiency comparison for SVG detection between PI and Seurat, SpatialDE, SPARK-X, and SINFONIA. **b**, Efficiency comparison for spatial domain identification between PNN and SpaGCN, BASS, SpaceFlow, and STAGATE.

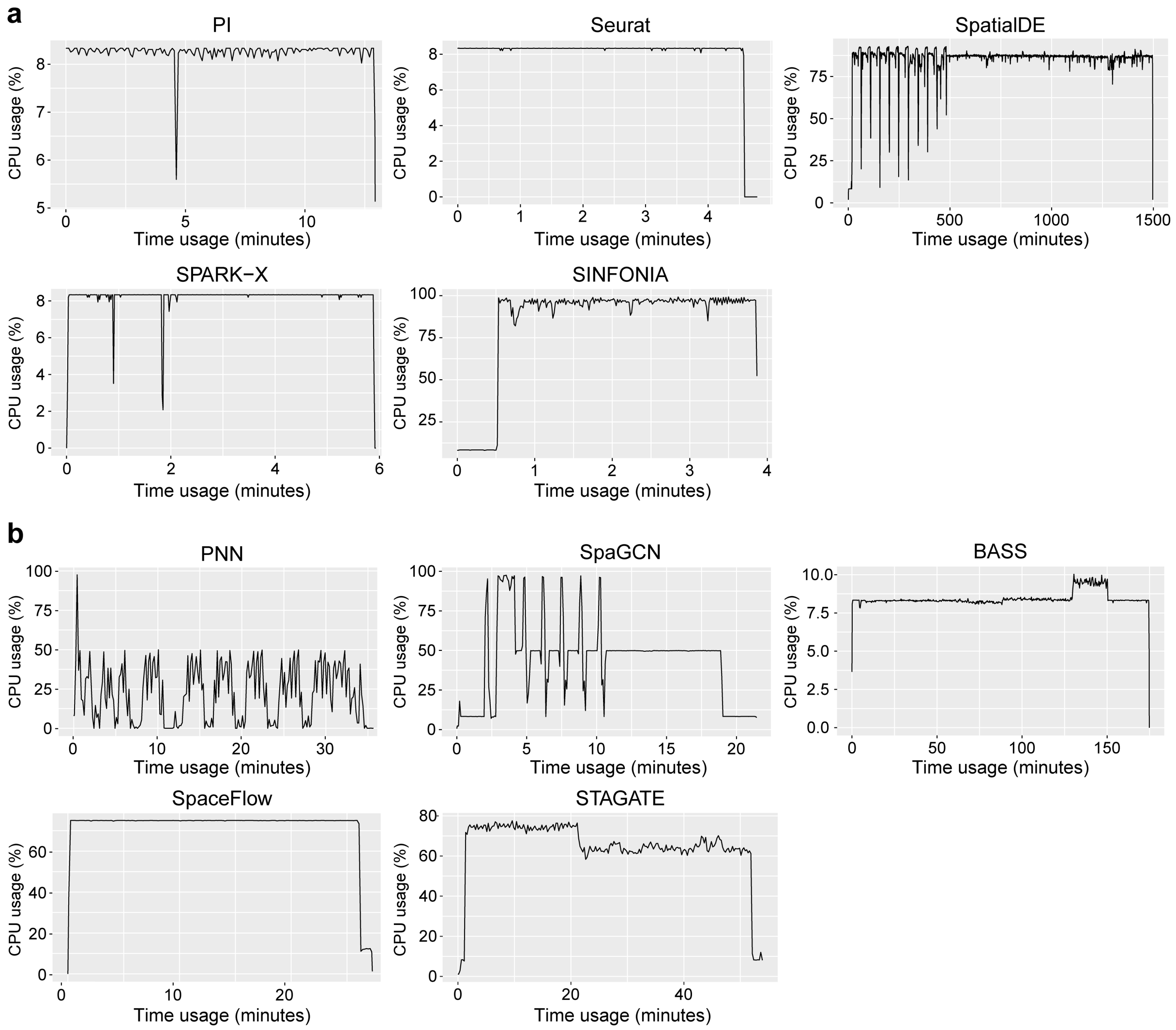

Supplementary Figure 36. Scalability analysis of SVG identification and domain segmentation methods. The simulated dataset[12], comprising 100 genes and cell numbers ranging from hundreds to millions, were utilized to evaluate the scalability of SVG identification (**a**) and domain segmentation (**b**) methods. Real datasets, encompassing adult human heart tissue ST dataset[15] and mouse complex tissues Slide-seq dataset[16], were employed to assess the scalability of SVG identification (**c, d**) and domain segmentation (**e, f**) methods, respectively. The evaluation of scalability includes running time and memory requirements.

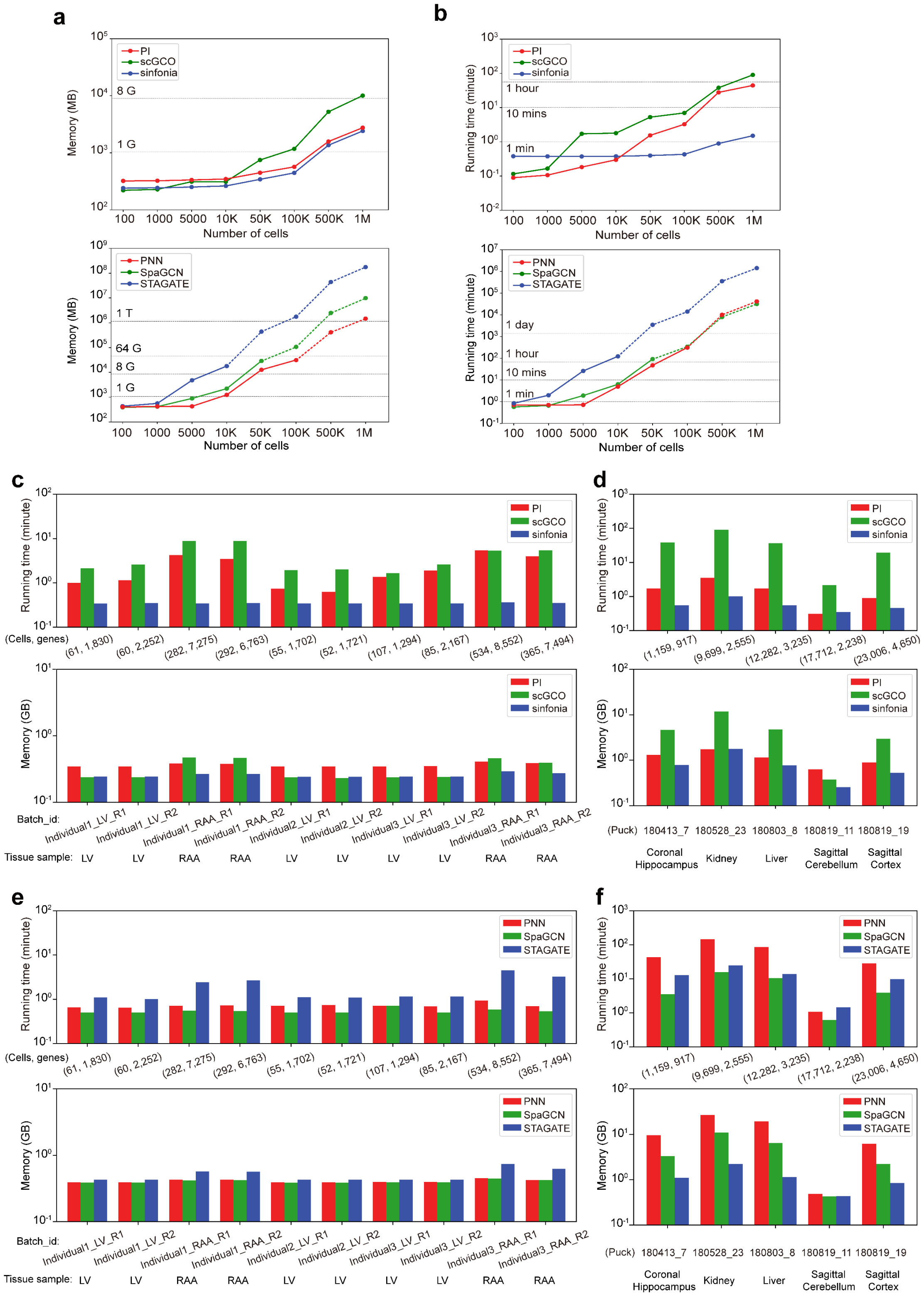

References:

1. Wolf, F. A., Angerer, P. & Theis, F. J. SCANPY: large-scale single-cell gene expression data analysis. *Genome Biol.* **19**, 15 (2018).

2. Pham, D. *et al.* stLearn: integrating spatial location, tissue morphology and gene expression to find cell types, cell-cell interactions and spatial trajectories within undissociated tissues. Preprint at https://doi.org/10.1101/2020.05.31.125658 (2020).

3. Dries, R. *et al.* Giotto: a toolbox for integrative analysis and visualization of spatial expression data. *Genome Biol.* **22**, 78 (2021).

4. Zhao, E. *et al.* Spatial transcriptomics at subspot resolution with BayesSpace. *Nat. Biotechnol.* **39**, 1375–1384 (2021).

5. Hu, J. *et al.* SpaGCN: Integrating gene expression, spatial location and histology to identify spatial domains and spatially variable genes by graph convolutional network. *Nat. Methods* **18**, 1342–1351 (2021).

6. Ren, H., Walker, B. L., Cang, Z. & Nie, Q. Identifying multicellular spatiotemporal organization of cells with SpaceFlow. *Nat. Commun.* **13**, 4076 (2022).

7. Dong, K. & Zhang, S. Deciphering spatial domains from spatially resolved transcriptomics with an adaptive graph attention auto-encoder. *Nat. Commun.* **13**, 1739 (2022).

8. Li, Z. & Zhou, X. BASS: multi-scale and multi-sample analysis enables accurate cell type clustering and spatial domain detection in spatial transcriptomic studies. *Genome Biol.* **23**, 168 (2022).

9. Hao, Y. *et al.* Integrated analysis of multimodal single-cell data. *Cell* **184**, 3573-3587.e29 (2021).

10. Svensson, V., Teichmann, S. A. & Stegle, O. SpatialDE: identification of spatially variable genes. *Nat. Methods* **15**, 343–346 (2018).

11. Zhu, J., Sun, S. & Zhou, X. SPARK-X: non-parametric modeling enables scalable and robust detection of spatial expression patterns for large spatial transcriptomic studies. *Genome Biol.* **22**, 184 (2021).

12. Zhang, K., Feng, W. & Wang, P. Identification of spatially variable genes with graph cuts. *Nat. Commun.* **13**, 5488 (2022).

13. Jiang, R., Li, Z., Jia, Y., Li, S. & Chen, S. SINFONIA: Scalable Identification of Spatially Variable Genes for Deciphering Spatial Domains. *Cells* **12**, 604 (2023).

14. Maynard, K. R. *et al.* Transcriptome-scale spatial gene expression in the human dorsolateral prefrontal cortex. *Nat. Neurosci.* **24**, 425–436 (2021).

15. Asp, M. *et al.* Spatial detection of fetal marker genes expressed at low level in adult human heart tissue. *Sci. Rep.* **7**, 12941 (2017).

16. Rodriques, S. G. *et al.* Slide-seq: A scalable technology for measuring genome-wide expression at high spatial resolution. *Science* **1463–1467**, 6 (2019).

17. Codeluppi, S. *et al.* Spatial organization of the somatosensory cortex revealed by osmFISH. *Nat. Methods* **15**, 932–935 (2018).

18. Fu, H. *et al.* Unsupervised Spatially Embedded Deep Representation of Spatial Transcriptomics. Preprint at https://doi.org/10.1101/2021.06.15.448542 (2021).

19. Stickels, R. R. *et al.* Highly sensitive spatial transcriptomics at near-cellular resolution with Slide-seqV2. *Nat. Biotechnol.* **39**, 313–319 (2021).

20. Lohoff, T. *et al.* Integration of spatial and single-cell transcriptomic data elucidates mouse organogenesis. *Nat. Biotechnol.* **40**, 74–85 (2022).

21. Cheng, A., Hu, G. & Li, W. V. Benchmarking cell-type clustering methods for spatially resolved transcriptomics data. *Brief. Bioinform.* **24**, bbac475 (2023).

22. Sunkin, S. M. *et al.* Allen Brain Atlas: an integrated spatio-temporal portal for exploring the central nervous system. *Nucleic Acids Res.* **41**, D996–D1008 (2012).
