# Supplementary material for: PROST: quantitative identification of spatially variable genes and domain detection in spatial transcriptomics
Source: Nat Commun. 2024 Jan 18;15:600. doi: 10.1038/s41467-024-44835-w (PMC10796707; doi:10.1038/s41467-024-44835-w)
Supplement: Supplementary file 3 — Reporting Summary [file 41467_2024_44835_MOESM3_ESM.pdf]

nature portfolio

Corresponding author(s): Zhonghui Tang

Last updated by author(s): Aug 20, 2023

Reporting Summary

Nature Portfolio wishes to improve the reproducibility of the work that we publish. This form provides structure for consistency and transparency in reporting. For further information on Nature Portfolio policies, see our Editorial Policies and the Editorial Policy Checklist.

Statistics

For all statistical analyses, confirm that the following items are present in the figure legend, table legend, main text, or Methods section.

n/a	Confirmed	
[ ]	[x]	The exact sample size (*n*) for each experimental group/condition, given as a discrete number and unit of measurement
[x]	[ ]	A statement on whether measurements were taken from distinct samples or whether the same sample was measured repeatedly
[ ]	[x]	The statistical test(s) used AND whether they are one- or two-sided<br>*Only common tests should be described solely by name; describe more complex techniques in the Methods section.*
[x]	[ ]	A description of all covariates tested
[ ]	[x]	A description of any assumptions or corrections, such as tests of normality and adjustment for multiple comparisons
[ ]	[x]	A full description of the statistical parameters including central tendency (e.g. means) or other basic estimates (e.g. regression coefficient) AND variation (e.g. standard deviation) or associated estimates of uncertainty (e.g. confidence intervals)
[ ]	[x]	For null hypothesis testing, the test statistic (e.g. *F*, *t*, *r*) with confidence intervals, effect sizes, degrees of freedom and *P* value noted<br>*Give P values as exact values whenever suitable.*
[x]	[ ]	For Bayesian analysis, information on the choice of priors and Markov chain Monte Carlo settings
[x]	[ ]	For hierarchical and complex designs, identification of the appropriate level for tests and full reporting of outcomes
[ ]	[x]	Estimates of effect sizes (e.g. Cohen's *d*, Pearson's *r*), indicating how they were calculated

Our web collection on statistics for biologists contains articles on many of the points above.

Software and code

Policy information about availability of computer code

Data collection	The data collection is stated in the method part of the manuscript. No additional software was used for the data collection process.
Data analysis	PROST is available on Github https://github.com/Tang-Lab-super/PROST. PROST package is built upon python 3.7 and requires dependencies, including anndata v0.7.6, h5py v3.7.0, leidenalg v0.9.0, natsort v8.2.0, numba v0.56.3, numpy v1.21.6, opencv-python v4.6.0.66, pandas v1.3.5, scanpy v1.8.2, scikit-image v0.19.2, scikit-learn v0.24.2, scipy v1.7.3, torch v1.8.1.

For manuscripts utilizing custom algorithms or software that are central to the research but not yet described in published literature, software must be made available to editors and reviewers. We strongly encourage code deposition in a community repository (e.g. GitHub). See the Nature Portfolio guidelines for submitting code & software for further information.

Data

Policy information about availability of data

All manuscripts must include a data availability statement. This statement should provide the following information, where applicable:

- Accession codes, unique identifiers, or web links for publicly available datasets
- A description of any restrictions on data availability
- For clinical datasets or third party data, please ensure that the statement adheres to our policy

All data analyzed in this study downloaded in raw form from the original publications. (1) The 10x Visium DLPFC data is available at LIBD (http://research.libd.org/spatialLIBD); (2) The other datasets generated by 10x Visium platform are available at the 10x Genomics website (https://support.10xgenomics.com/spatial-gene-expression/datasets); (3) The processed Stereo-seq data from mouse olfactory bulb tissue is accessible at SEDR (https://github.com/JinmiaoChenLab/

March 2021

SEDR_analyses); (4) The Slide-seqV2 datasets are available at the Broad Institute Single Cell Portal (https://singlecell.broadinstitute.org/single_cell/study/SCP815/highly-sensitive-spatial-transcriptomics-at-near-cellular-resolution-with-slide-seqv2); (5) Processed SeqFISH mouse embryogenesis dataset with segmentation information and associate metadata are available at https://crukci.shinyapps.io/SpatialMouseAtlas/.

Human research participants

Policy information about studies involving human research participants and Sex and Gender in Research.

Reporting on sex and gender	NA
Population characteristics	NA
Recruitment	NA
Ethics oversight	NA

Note that full information on the approval of the study protocol must also be provided in the manuscript.

Field-specific reporting

Please select the one below that is the best fit for your research. If you are not sure, read the appropriate sections before making your selection.

- [x] Life sciences
- [ ] Behavioural & social sciences
- [ ] Ecological, evolutionary & environmental sciences

For a reference copy of the document with all sections, see nature.com/documents/nr-reporting-summary-flat.pdf

Life sciences study design

All studies must disclose on these points even when the disclosure is negative.

Sample size	No sample size calculation was performed. All data used in this study were collected from public resources and used to demonstrate the functionalities and performance of PROST.
Data exclusions	We performed quality control of spatial resolved transcriptomics data based on the commonly used and pre-established criteria in this field.
Replication	NA
Randomization	NA
Blinding	All analysis were based on the published data which have been studied in their original publications. Therefore, blinding from investigators is not possible when we reanalyzed the data.

Reporting for specific materials, systems and methods

We require information from authors about some types of materials, experimental systems and methods used in many studies. Here, indicate whether each material, system or method listed is relevant to your study. If you are not sure if a list item applies to your research, read the appropriate section before selecting a response.

Materials & experimental systems

n/a	Involved in the study	
☒	☐	Antibodies
☒	☐	Eukaryotic cell lines
☒	☐	Palaeontology and archaeology
☒	☐	Animals and other organisms
☒	☐	Clinical data
☒	☐	Dual use research of concern

Methods

n/a	Involved in the study	
☒	☐	ChIP-seq
☒	☐	Flow cytometry
☒	☐	MRI-based neuroimaging
